# Evaluating the Impact of Virtual Reality on the Behavioral and Psychological Symptoms of Dementia and Quality of Life of Inpatients With Dementia in Acute Care: Randomized Controlled Trial (VRCT)

**DOI:** 10.2196/51758

**Published:** 2024-01-30

**Authors:** Lora Appel, Eva Appel, Erika Kisonas, Samantha Lewis-Fung, Susanna Pardini, Jarred Rosenberg, Julian Appel, Christopher Smith

**Affiliations:** 1 School of Health Policy and Management Faculty of Health York Universtiy Toronto, ON Canada; 2 OpenLab Toronto Rehabilitation Institute University Health Network Toronto, ON Canada; 3 Michael Garron Hospital Toronto, ON Canada; 4 University of Padua Padua Italy; 5 Ted Rogers School of Management Toronto Metropolitan University Toronto, ON Canada

**Keywords:** digital therapies, nonpharmacological, cognitive impairment, behavioral and psychological symptoms of dementia, BPSDs, randomized controlled trial, virtual reality

## Abstract

**Background:**

Virtual reality (VR) is increasingly considered a valuable therapeutic tool for people with dementia. However, rigorous studies are still needed to evaluate its impact on behavioral and psychological symptoms of dementia (BPSDs) and quality of life (QoL) across care settings.

**Objective:**

The primary aim of this study was to evaluate the impact of VR therapy on managing BPSDs, falls, length of stay, and QoL in inpatients with dementia admitted to an acute care hospital. The secondary aim was to evaluate the intervention’s feasibility in terms of acceptability, safety, and patient experience.

**Methods:**

A prospective, open-label, mixed methods, randomized controlled clinical trial was conducted between April 2019 and March 2020. A total of 69 participants (aged ≥65 years with a diagnosis of dementia and who did not meet the exclusion criteria) were randomly assigned to either the control (n=35, 51%) or VR (n=34, 49%) arm. Participants in the experimental (VR) arm were visited by a researcher and watched 360° VR films on a head-mounted display for up to 20 minutes every 1 to 3 days, whereas individuals in the control arm received standard of care. Instances of daily BPSDs and falls were collected from nurses’ daily notes. QoL was measured through semistructured interviews and the Quality of Life in Late-Stage Dementia scale. Structured observations and semistructured interviews were used to measure treatment feasibility. The primary outcomes were analyzed at a 95% significance level based on the intention-to-treat method.

**Results:**

VR therapy had a statistically significant effect on reducing aggressiveness (ie, physical aggression and loud vociferation; *P*=.01). Substantial impact of VR therapy was not found for other BPSDs (eg, apathy), falls, length of stay, or QoL as measured using the Quality of Life in Late-Stage Dementia scale. The average VR therapy session lasted 6.8 (SD 6.6; range 0-20) minutes, and the intervention was overall an acceptable and enjoyable experience for participants. No adverse events occurred as a result of VR therapy.

**Conclusions:**

Immersive VR therapy appears to have an effect on aggressive behaviors in patients with dementia in acute care. Although the randomized controlled trial was stopped before reaching the intended sample size owing to COVID-19 restrictions, trends in the results are promising. We suggest conducting future trials with larger samples and, in some cases, more sensitive data collection instruments.

**Trial Registration:**

ClinicalTrials.gov NCT03941119; https://clinicaltrials.gov/study/NCT03941119

**International Registered Report Identifier (IRRID):**

RR2-10.2196/22406

## Introduction

### Managing Behavioral and Psychological Symptoms of Dementia in Acute Care

Behavioral and psychological symptoms of dementia (BPSDs) constitute a heterogeneous cluster of signs and symptoms [1,2] that are present in up to 90% of people with dementia [3] and are difficult to manage [4]. In acute care settings, approximately 75% of inpatients with dementia display BPSDs during their stay [5] as stress and hyperstimulation from being in a hospital environment are known exacerbating factors [6].

BPSDs are linked to key acute care outcomes, including falls [7] and longer length of stay (LoS) [8-10], fueling a cycle of decline. Longer stays can result in negative consequences such as declines in cognition and function [11], which in turn are associated with a significantly higher risk of hospitalization and institutionalization [12-19] and increased health care spending [8,20,21]. Moreover, aggression and agitation, the most frequently displayed BPSDs in this setting [9], mediate symptoms of caregiver burden and decrease quality of life (QoL) and well-being in people with dementia and caregivers alike [8,21].

The current means to manage BPSDs in acute care are insufficient and, at times, pose ethical challenges. Pharmacological therapy, often in the form of atypical antipsychotics, remains the most common treatment for BPSDs [22]. Even with newer drugs in this class, clinical outcomes are poor, and undesired side effects such as cognitive worsening, confusion [22], and extrapyramidal symptoms are prevalent [23]. Other common alternatives for managing BPSDs include using physical barriers such as wrist or ankle restraints, alarms, locks, Buxton chairs, and tethers. Unfortunately, these approaches have a wide range of negative consequences for psychological and physical functioning in people with dementia (ie, lethargy, cognitive decline, anxiety, distress, aggression, increased risk of falls, and pressure sores) [24,25].

For some time, guidelines for managing BPSDs have recommended using person-centered [26-28] psychosocial nonpharmacological interventions as a first-line approach [29,30], and qualitative studies exploring the in-hospital experiences of people with dementia have echoed the need for improved ward-based activity services [31,32]. Some examples considered to be gold standards include the use of physical presence and therapeutic touch, recreational or social activities, behavioral interventions, individual and group psychological or psychosocial interventions, environmental interventions, daily routines, music-based therapies [33,34], or combinations of these [35,36]. Notably, a recent review concluded that nonpharmacological interventions are a safer alternative to pharmacological interventions and can effectively reduce certain BPSDs [37]. Exposure to natural stimuli (eg, views of trees, flowers, or outdoor environments) is a nonpharmacological intervention that has been consistently shown to (1) enhance well-being and QoL; (2) reduce depression, anxiety, agitation or aggression, and the use of analgesics; and (3) decrease hospital LoS for inpatients [38-41]. A landmark study by Ulrich [38] showed that patients who had a window with a view of nature rather than a brick wall had reduced use of analgesics and a shortened LoS in hospital. Since then, several studies have shown that exposure to nature reduces agitation or aggression and promotes feelings of joy and relaxation in people with dementia [39-41]. Even the presence of an ornamental plant in the hospital room has significant positive effects on stress, anxiety, fatigue, and systolic blood pressure in patients recovering from surgery [42].

However, providing people with dementia with exposure to nature is challenging in acute care settings. A key difficulty is that, when individuals with dementia are admitted to hospital, they leave their beds infrequently because of existing mobility challenges [43] or hospital-acquired complications [44]. Although assistance with ambulation is both beneficial for maintaining preadmission functional status [44] and perceived by staff as important for safety [31,45], patients’ basic needs often go unmet because of staff workload [31].

### Virtual Reality Interventions

One way in which people who are unable to engage directly with a natural environment can still be exposed to nature is by using virtual reality (VR) technology. VR uses a head-mounted display (HMD) to generate simulated immersive experiences that create a sense of presence. The sensation of being present in a different physical place is achieved by simultaneously stimulating multiple senses (typically vision and hearing), obscuring the real environment and replacing it with an artificial “virtual” environment [46].

There has been a recent increase in interest in the therapeutic use of VR with older adults in general [47-50], with most of the studies investigating the impact of VR therapy on well-being and BPSDs in people with dementia published since 2018 [51]. VR interventions for people with dementia constitute a cost-effective, noninvasive, and ethically acceptable alternative for managing BPSDs by redirecting the patient’s attention within a physically safe environment [52,53]. To date, these interventions have typically used music and sensory and mental stimulation with the aim of improving cognitive functions (attention, executive function, and visual and verbal memory), psychological symptoms (agitation, depression, anxiety, and apathy) [46,50,54], and the retrieval of autobiographical memories [51,52,55]. It has been suggested that VR therapy can be deployed in place of pharmacological therapy to improve well-being and QoL [56]; however, further research is necessary to establish its effectiveness for this purpose [51].

Our prior work has shown that it is feasible for frail older adults with varying degrees of physical mobility and cognitive impairment to wear a VR HMD and engage with natural VR environments in a community care setting [52,57]. These findings are consistent with those of other research showing that exposure to a VR forest resulted in increased feelings of pleasure and greater levels of alertness in people with dementia [58]. A growing number of studies have also shown significant positive results in using VR to manage acute pain in hospitalized patients without cognitive impairment [59]. To summarize, although VR offers an apparent solution for providing access to natural scenery to inpatients with limited mobility, its use with inpatients with dementia remains largely unexplored.

The methodology and aims of this randomized controlled trial (RCT) were directly informed by our previous studies: (1) a multisite feasibility study in 2 rehabilitation facilities (a long-term care facility and a dementia day program center) and (2) a pilot study conducted in the hospitalist medicine department of Michael Garron Hospital (MGH). The feasibility study recruited 66 outpatients with cognitive, sensory, and mobility impairments and demonstrated that it is safe and feasible to administer VR therapy in this population using an HMD [57]. The pilot study recruited 10 inpatients with dementia to validate the proposed RCT study protocol (processes, methods, recruitment strategy, resource requirements, and timelines) [52,53]. In both studies, participants reported on average more positive than negative feelings after experiencing nature scenarios, tolerated the VR hardware, and were able to physically explore the 360° virtual environments by turning their heads.

### Aims and Hypothesis

To the best of our knowledge, an in-depth analysis of VR’s impact on people with dementia has not yet been carried out in this setting. The main objectives of this RCT were to evaluate the impact of VR therapy on BPSDs and associated patient outcomes during the hospital stay. Our secondary objectives were to report on the acceptability, safety, experience, and satisfaction with VR therapy among inpatients with dementia. Specifically, we predicted that, for older adults with dementia admitted to an acute care hospital, (1) VR therapy would help manage BPSDs (ie, decrease anxiety, aggression, depression, violent behaviors, and incidents of wandering) and may decrease the number of incidents that require restraints, LoS, and the number of falls; (2) VR therapy would improve QoL (operationalized through validated instruments that measure QoL for people with dementia); and (3) VR therapy is safe and feasible (in terms of acceptability and patient experience) to administer.

## Methods

### Study Population

Inpatients aged >65 years with a documented diagnosis of dementia were recruited from MGH in the hospitalist or internal medicine ward. Data collection occurred between April 29, 2019, and March 13, 2020. The study was temporarily stopped under an MGH Research Ethics Board directive on March 26, 2020, when the first wave of COVID-19 peaked in Canada. Subsequently, recruitment was permanently ceased owing to the ongoing pandemic and restrictions on research, specifically with this vulnerable population.

### Study Design

This study was an open-label RCT conducted at MGH, a community teaching hospital located in Toronto, Ontario, in collaboration with OpenLab, an innovation center at the University Health Network. The clinical trial protocol was registered at ClinicalTrials.gov (NCT03941119).

Individuals were randomly assigned to either the VR arm or the control arm. During their hospital stay, both groups received a visit from the research coordinator (RC) every 24 to 72 hours to complete a QoL assessment. Patients in the VR arm then participated in a VR therapy session for up to 20 minutes. This dosage regimen was informed by previous studies in which VR was used to promote well-being and manage BPSDs in people with dementia [60], as well as by other nonpharmacological interventions in acute care [51]. Those in the control arm were not exposed to VR therapy and otherwise followed the standard of care (ie, medical treatment for the presenting issue and management of any psychological and behavioral symptoms based on standard guidelines for hospitals in Ontario).

### Ethical Considerations

Approval for research involving human participants was received from the MGH Research Ethics Board (782-1812-Mis-332) on January 14, 2019.

The consent process followed Ontario’s legislation [61], and the hospital provided a brochure [62] to help patients and families understand the different roles involved in decision-making. Any previously documented capacity assessments were respected. For potential participants with no record of capacity assessment, the capacity to provide informed consent was obtained as per the study site protocol. If the patient had a documented capacity assessment determining that they were able to provide informed consent, the RC approached them in person to ask about their interest in the study. In cases in which a capacity assessment was required or a capacity assessment determined that the patient was unable to provide informed consent, the substitute decision-maker (SDM) was contacted as early into the patient’s stay as possible. To ensure patient assent in all cases possible, informed SDM consent was provided with the patient present. The RC provided a copy of the informed consent or assent form and an informational study brochure (Multimedia Appendices 1-3) for review to all interested participants and their SDMs. For participants in the VR arm, the RC obtained verbal or physical assent (body language indicating that the person agreed with the procedure or activity) from the participant immediately preceding any VR therapy session.

Data were anonymized and deidentified at the time of collection (ie, linked only to the study ID). Participants were not offered an honorarium or any form of compensation for taking part in the study. The patients provided written informed consent to allow their image to be published.

### Procedures

#### Screening

All new patient admissions to the hospitalist or internal medicine department at MGH were screened daily, excluding weekends and statutory holidays, using the hospital’s electronic medical record (EMR) system. Inclusion criteria required that participants be aged ≥65 years, have a documented diagnosis of dementia, and be admitted as inpatients at MGH. Patients were excluded if they had open facial wounds (sutured lacerations exempted), a history of seizures or epilepsy, a pacemaker, head trauma or stroke leading to their current admission, cervical conditions that would make use of a VR headset unsafe, or alcohol-related dementia or Wernicke-Korsakoff syndrome or were admitted to the intensive care unit or adult mental health inpatient service. Patients were also excluded if they had participated in the current RCT during a previous hospitalization or were readmitted <30 days after discharge. Finally, patients with no contactable SDM or who had a Public Guardian and Trustee as SDM were also excluded. Before recruitment, potential participants were unknown to the RC who administered the experimental treatment.

#### Randomization

After obtaining informed consent, the participants were randomized at a 1:1 allocation ratio to either the VR arm or the control arm. Random allocation to 1 of the 2 study arms was achieved using a digital tool performing Maximally-Tolerated-Imbalance randomization, a method designed to mitigate selection biases, simultaneously lowering allocation predictability and protecting against chronological bias [63]. After randomization, the participants, caregivers, and research personnel were not blind to treatment allocation given the nature of the intervention. The ward staff were blinded to treatment allocation to the greatest extent possible. Participants and caregivers were asked not to discuss their arm allocation with the ward staff. The sessions were scheduled to accommodate the daily activities of patients and their care teams with respect to their acute care needs and were facilitated by a research team member who was trained in dementia care.

#### Sample Size

With no previous experiments from which to obtain expected significant differences or sample SDs, we calculated our sample size based on our feasibility study [57], which collected pretest-posttest changes in emotional state using a modified version of the State-Trait Anxiety Inventory tool. Our feasibility study captured pretest-posttest State-Trait Anxiety Inventory results by category (called “Emotional Modality”). We computed pooled sample SDs using the aggregated results to obtain the required sample sizes for a significance level of 95% and a power of 80%. The results for the categories considered “positive” (eg, calm, relaxed, and content) had a mean estimated sample size of 181. Thus, at an assumed attrition rate of 20%, it was determined that 225 participants would need to be recruited to obtain a final sample of 180 patients.

### Intervention

#### Hardware

**Figure 1:**
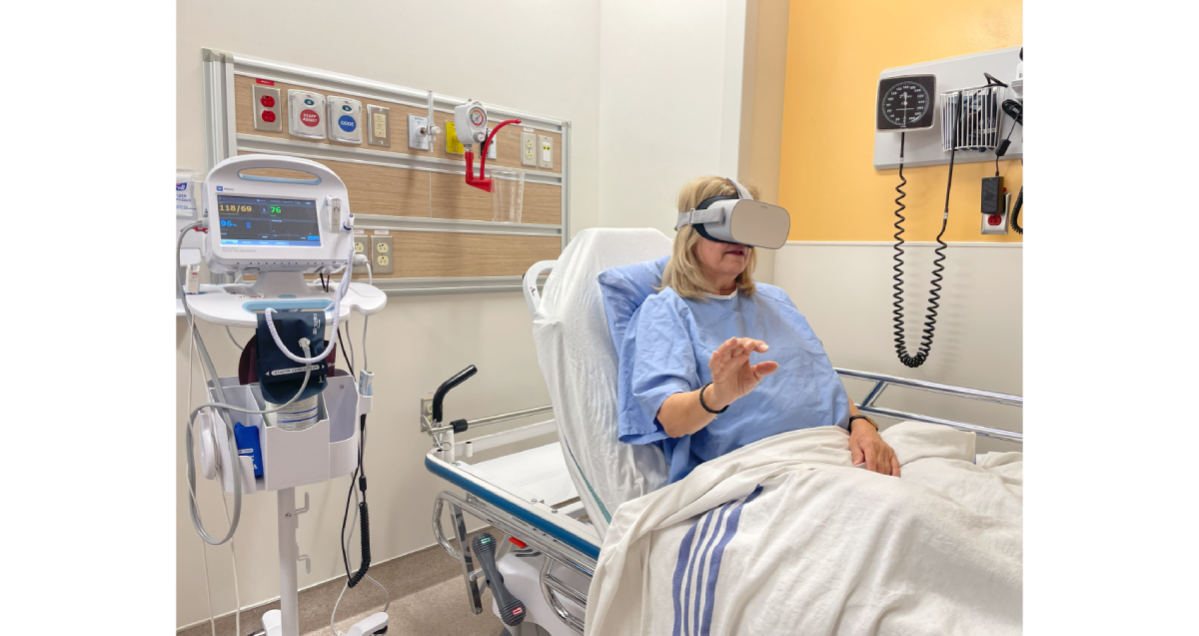
Photo of a patient wearing a virtual reality head-mounted display (without external headphones).

The Oculus Go (Meta Reality Labs), a stand-alone mobile VR HMD system (discontinued on June 23, 2020), was selected based on its relative affordability and portability [64] and reduced likelihood of inducing simulator sickness owing to its low motion latency. This HMD requires no external hardware (ie, sensors to track head movement). It has built-in headphones, eliminating a number of difficulties raised by using external headphones (ie, cumbersome to mount and fit during VR therapy and increased time fulfilling hospital infection prevention and control requirements). External headphones (Sennheiser HD 221) were available for use if participants chose to use them or if the RC determined that they would be particularly helpful in certain situations (ie, when a participant was very hard of hearing or in a noisy environment). Figure 1 depicts an older patient wearing a VR HMD without external headphones (informed consent was provided by the patient for the publication of this photograph).

#### Software

**Figure 2:**
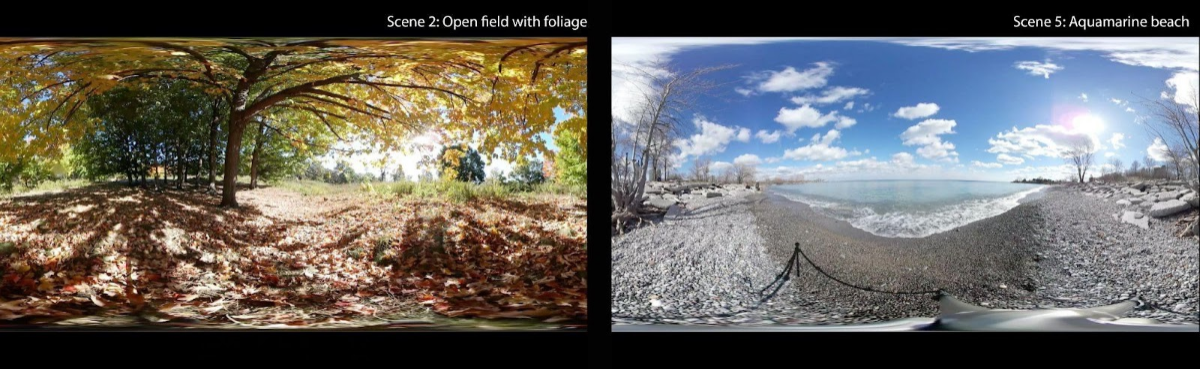
2D screen capture of nature scenes from 2 of the 360° films shown to patients.

A library of short 360° films was assembled based on previous studies and expert consultation. The VR films were intentionally chosen to be calming in distinctive ways as supported by the literature on nature visualization and well-being. By varying the (1) length of the films (1-, 3-, or 5-min clips), (2) types of natural elements (greenery, sky, and water), (3) distance of view (close-ups and vistas), (4) motion (flowing water and wind in trees), (5) sounds (leaves rustling and music from a live band), and (6) presence of different elements (eg, people in social settings, remote nature scenery, and urban sites), the content provided a wide range of features that could be collaboratively selected by the participant, caregiver, or health care provider for each individual in the VR arm. The scenes were designed specifically for people with dementia. First-person movement was limited to reduce the potential for simulator sickness, as was the use of excessive sound, light, or movement to avoid overstimulation. Figure 2 displays a 2D screen capture of 2 of the 7 VR scenes shown during the study (scene 2, “Open Field with Foliage,” and scene 5, “Aquamarine Beach”). The films were accessed during the study sessions by the researcher from the VR HMD’s internal storage.

#### Session Protocol

This study used a mixed methods design. Measures included baseline demographics (diagnoses, cognitive impairment, delirium, and comorbidities) and factors related to the participants’ hospital care experience (falls with or without injury, LoS, and nurse documentation of daily BPSDs) collected from the hospital’s EMR. During their hospital stay, recruited patients were visited by the RC every 24 to 72 hours (1-3 days), who documented findings using a validated QoL scale, a standardized observational tool, semistructured interviews with participants and their caregivers, and qualitative observations. Any episodes of adverse events (such as vertigo, dizziness, nausea, neck pain, and headaches) were documented. Table 1 provides a comprehensive list of the outcomes of interest, their evaluation methods, and the frequency of collection.

Before any study session, depending on participant allocation (VR or control arm), the RC consulted ward staff to ensure that the participant was in a stable condition for VR therapy and had no scheduled treatment or tests in the following hour (see Multimedia Appendix 4 for the presession checklist). As per the study geriatrician’s recommendations, if the participant was asleep upon arrival, the RC attempted to gently wake up the patient by saying their name a few times or returned later in the day if the patient remained asleep. If able and willing to sit, the participant was then encouraged to sit upright. Otherwise, they participated in the session lying down with the bed tilted to an upright position. If a participant required assistance to move to an appropriate position, the RC contacted a nurse for assistance.

During the first visit, the RC conducted a baseline demographic survey with the participant and their informed caregiver (if present). At the beginning of each study session, the RC engaged the participant in at least 5 minutes of unstructured conversation followed by a brief semistructured interview regarding their well-being during their hospital stay and then completed the modified Quality of Life in Late-Stage Dementia (QUALID) scale (Multimedia Appendix 5). Participants in the control arm then continued per the current standard of care (ie, the study visit was complete at this juncture, with no exposure to VR therapy). For VR participants, the RC placed the VR headset on the participant’s head, assisted with adjusting the straps for a comfortable fit, and then observed while they watched 360° VR films for up to 20 minutes. During the intervention, the RC recorded observational notes pertaining to facial expressions, body language, and verbal feedback based on a standardized VR observational tool that the research team designed and used in previous VR studies (Multimedia Appendix 6; this tool has since been further refined [65]). After the VR therapy intervention, the RC conducted a semistructured interview with the participant and informal caregiver (if present) to gather their feedback on their experience and aspects related to the acceptability of VR therapy and confirmed the participant’s willingness to take part in additional sessions during their hospital stay.

**Table 1:** Evaluation tools and methods by outcome of interest.

			Outcomes measured		Method or data collection tool
**Primary objectives: VR^a^ and control arms**					
	Baseline demographics^b^	Gender, age, primary language, highest level of education, current living state, relationship status, major visual and auditory conditions, use of glasses and hearing aids, head mobility, body mobility, mobility aids, dementia diagnosis, delirium severity, Charlson Comorbidity Index, MMSE^c^, and MoCA^d^		EMR^e^ Baseline demographic survey with participant and caregiver (if present)	
	Changes in BPSDs^f,g^	NPI-10^h^ cluster (delusions, hallucinations, agitation, depression, anxiety, apathy, irritability, euphoria, disinhibition, and aberrant motor behavior), aggression cluster (aggression, screaming or loud vocalization, restraints applied, security intervened, and bedside sitter), and wandering cluster		Key terms from daily notes in the EMR on patient symptoms were categorized into 3 clusters for analysis: NPI-10–like Violence Wandering	
	Falls^g^	Fall precautions, falls with injury, and falls without injury		EMR	
	Length of stay^i^	Length of stay (in min)		EMR	
	Quality of life^j^	Interacts with others, smiles, appears sad, cries, demonstrates or verbalizes discomfort, enjoys touching or being touched, appears emotionally calm and comfortable, appears physically uncomfortable, enjoys eating, and sleeps well		In-Hospital Quality of Life Observational Scale—adapted from the QUALID^k^ scale (includes semistructured interview)	
**Secondary objectives: VR group only**					
	VR acceptability^l^	Length of time viewing VR; pain, weight of device, comfort, and ability to wear glasses with device; and quality of visuals and sound		Structured observations Semistructured interview	
	VR safety^l^	Nervous or anxious, confused or disoriented, nauseous, medical device interaction (eg, glasses and hearing aids), and adverse events		Structured observations Semistructured interview	
	VR experience^l^	Enjoyment, relaxation, vocalizations, engagement, and reminiscence; opinions or preferences regarding content; and willingness to try VR again and recommend VR to a friend		Standardized VR observation tool Semistructured interview	

^a^VR: virtual reality.

^b^Collected during first study session.

^c^MMSE: Mini-Mental State Examination.

^d^MoCA: Montreal Cognitive Assessment.

^e^EMR: electronic medical record.

^f^BPSD: behavioral and psychological symptom of dementia.

^g^Reported daily (data set extracted once at discharge).

^h^NPI-10: Neuropsychiatric Inventory.

^i^Reported once at discharge.

^j^Conducted or assessed at each study session.

^k^QUALID: Quality of Life in Late-Stage Dementia.

^l^Conducted or assessed at each VR therapy session.

### Analysis

#### Overview

Statistical analyses were conducted using the Minitab statistical package (Minitab, LLC) [66]. The Anderson-Darling test of normality was run for all variables to determine whether to use statistical regression or a nonparametric test. Linear regression was used for outcomes with normally distributed values (Anderson-Darling *P*>.05), with arm assignment (VR vs control) as the main factor. To determine whether differences could be attributed to the treatment effect rather than to individual characteristics, other factors included in the regression analyses were whether the participant had had VR therapy sessions, age group, gender, cognitive level, and dementia type (see Multimedia Appendix 7 for a complete description of the participants’ baseline characteristics). Only data from the QUALID showed a normal distribution. All other measures were found to not be normally distributed (Anderson-Darling *P*<.05). The nonparametric Mood median test was chosen to measure differences in the distribution of the outcomes between the control and VR arms for these variables.

#### Primary Outcome Measures

##### Overview

The primary outcomes in this study pertained to the effects of VR therapy on BPSDs and other critical acute care outcomes. Data were analyzed at a 95% significance level based on the intention-to-treat (ITT) and per-protocol analysis methods [67-69]. Outcomes that were collected regularly throughout the study (BPSDs, falls, and QoL) were measured as the difference (“after” minus “before”) between the means of outcomes after and before the treatment, whereby we defined these as follows:

1. Before: the mean number of events or mean score up to (and including) the first session with the RC
2. After: the mean number of events or mean score after the first session with the RC, up to (and including) the last interaction with the RC or discharge

##### BPSD Outcome Measure

Data on BPSDs were collected from nurses’ notes in the EMR that reported daily patient symptoms. Key terms from the nurses’ notes were then categorized and analyzed based on the following predetermined clusters: (1) the Neuropsychiatric Inventory (NPI-10)–like cluster [70,71] (delusions, hallucinations, agitation, depression, anxiety, apathy, irritability, euphoria, disinhibition, and aberrant motor behavior), (2) the aggression cluster (aggression, screaming or loud vocalization, restraints applied, security intervened, and bedside sitter), and (3) the wandering cluster. Each cluster was analyzed separately. The categorization and analysis were completed by 2 coders and reviewed by a third, all of whom were blinded to arm assignment. It should be noted that, for the NPI-10–like cluster, the scale itself was not administered. In practice, the NPI-10 is typically conducted as a periodic measurement at wide time intervals. Thus, the possibility of administering the instrument was deemed impractical in the pilot study that informed this research [53]. Instead, the measurements were taken daily by focusing on keywords in the nurses’ notes that corresponded to items described by the NPI-10 instrument.

##### Falls

Outcomes concerning falls were operationalized as the frequency of additional fall precautions applied, falls with injury, and falls without injury during the hospital stay as recorded in the EMR.

##### LoS Outcome Measure

The length of acute hospital stays was calculated as the difference in minutes between the discharge date/time and admission date/time as recorded in the EMR.

##### QoL Outcome Measure

QoL was measured using an adapted version of the QUALID scale [72]. This adapted scale (Multimedia Appendix 5) was administered by the RC at the beginning of each study session. The QUALID contains 11 items with total scores ranging from −22 to 22, with a higher score indicating a better in-hospital QoL. The scale was modified to include a brief semistructured interview in which participants were asked open-ended questions related to their in-hospital QoL and well-being. A 5-point visual scale was used to assist participants in answering the semistructured interview question (“How are you feeling today?”).

RC notes detailing participants’ responses to the semistructured interview questions were manually coded for overall sentiment (ie, indicating positive, negative, or neutral QoL or well-being at that moment) by 2 independent coders (SL-F and JA) who were blinded to arm assignment. Any discrepancies in coding were discussed by the 2 raters until an agreement was reached. Responses that were judged to be positive (eg, “Not bad. Everyone was pretty nice today”), neutral (eg, “So-so,”), and negative (eg, “Not too well.... Tough night, improving marginally”) were scored as 1, 0, and −1, respectively. Unrelated and off-topic responses in which the participant’s overall sentiment could not be clearly discerned (eg, “Yes” and “Is there someone there?”) were excluded from the analysis.

#### Secondary Outcome Measures

##### Overview

Through the secondary outcomes, we sought to investigate the safety, acceptability, and feasibility of administering VR therapy to inpatients with dementia in an acute care setting. The secondary outcomes were analyzed by session and only for patients in the VR arm who received VR therapy. Quantitative data such as session length, observational tool scores, and responses to closed-ended interview questions were analyzed using descriptive statistics. Inferential methods were not used to examine secondary outcomes because of the small sample size. Observational data from the first sessions versus any subsequent sessions were compared manually to determine whether there were any clear trends that would warrant analysis by participant versus by session. Descriptive statistics revealed no clear patterns of differences that would warrant separate analysis by participant as opposed to by session.

Responses to open-ended semistructured interview questions, RC observations, and participant and caregiver comments during the session were analyzed thematically using the NVivo qualitative data analysis computer software package (Lumivero) by EK, SL-F, and LA [73]. The constant comparative method [74,75] was used to systematically explore the qualitative data and corroborate and expand upon the results from quantitative measures. This method involved using an open coding process over several iterations, with the authors meeting at each stage to discuss and compare emerging themes and contradictions within different sections of the data until core categories emerged [75].

##### Acceptability of VR Therapy

Acceptability was operationalized through the total time spent viewing VR, reported and observed aspects of comfort, and adequacy of the stimuli. Participants in the VR arm were asked whether the VR HMD was painful, too heavy, or otherwise uncomfortable and whether the visual and auditory stimuli were sufficiently provided (ie, good resolution and good sound). Structured observations of acceptability were recorded during sessions by the RC, including reasons for stopping sessions early.

##### Safety of VR Therapy

Safety was operationalized as any adverse events reported in the EMR as well as any feelings of nervousness or anxiety, confusion or disorientation, or medical device interaction (ie, buzzing from hearing aids). Data were collected from the EMR and through RC observations of VR therapy sessions and postsession semistructured interviews with the participants.

##### Participant Experience During VR Therapy

Participants’ experiences during and immediately after VR therapy were assessed at each study session and operationalized through observations of their level of enjoyment, relaxation, engagement, and vocalizations, as well as the presence of reminiscence. The RC observed and rated participants’ reactions to VR therapy using the standardized VR observation tool (Multimedia Appendix 6) during VR therapy and recorded any other behavior suggesting a pleasant or unpleasant experience. A semistructured interview immediately after VR therapy was conducted that included open-ended questions on experience as described previously as well as willingness to try VR therapy again, willingness to recommend VR to a friend, and interest in purchasing a VR device. Opinions and preferences regarding the themes or content of the VR films were also collected.

## Results

### Demographics

Of the 3514 new admissions to acute care during the study period, 300 (8.54%) met the inclusion criteria. After contacting the SDMs of eligible participants and excluding patients who met the exclusion criteria (ie, discharged or deceased) or refused to use the VR equipment, a total of 77 patients with dementia were consented and enrolled in the study. Participants were randomized to the control arm with no VR therapy exposure (39/77, 51%) and the intervention arm receiving VR therapy once every 1 to 3 days (38/77, 49%).

**Figure 3:**
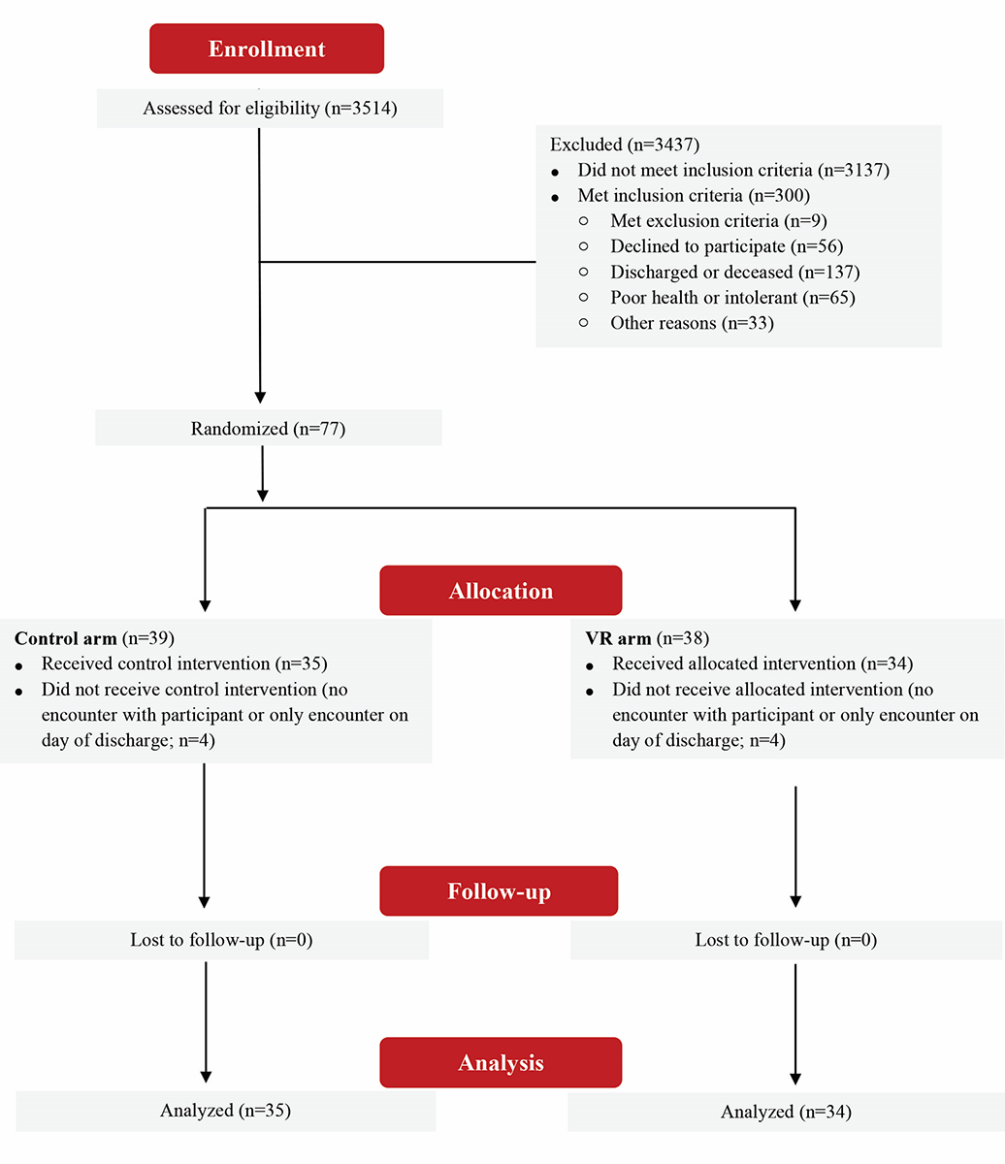
CONSORT (Consolidated Standards of Reporting Trials) diagram. Patient flow based on intention-to-treat method. VR: virtual reality.

From each of the 2 arms, 4 participants were excluded (4/39, 10% from the control arm and 4/38, 11% from the intervention arm) as there was no encounter with the participants to allow for meaningful comparison (ie, the only interaction occurred on the last day of the hospital stay), leaving 69 participants in total, with 35 (51%) in the control arm and 34 (49%) in the experimental VR therapy arm. In the VR arm, 85% (29/34) of the participants completed at least one session of VR therapy, whereas 15% (5/34) refused the VR and completed no VR therapy sessions. Figure 3 shows a CONSORT (Consolidated Standards of Reporting Trials) diagram of patient flow based on ITT.

Multimedia Appendix 7 describes the baseline demographics of all study participants (n=69) as reported in clinical documentation based on 3 groups: the control arm and 2 VR arms, “VRx arm: With VRx-therapy” and “VRx arm: No VRx-therapy.” Across arms, 65% (45/69) of the participants were female and belonged to the following age groups: 28% (19/69) were aged 65 to 79 years, 33% (23/69) were aged 80 to 89 years, and 39% (27/69) were aged 90 to 100 years. Over half of the participants had either a moderate (23/69, 33%) or severe (11/69, 16%) level of cognitive impairment, and nearly half either required assistance (7/69, 10%) or were unable (22/69, 32%) to sit. Vision and hearing aids were used by 62% (43/69) and 23% (16/69) of the participants, respectively.

To determine the possibility of a priori bias in the random assignment, a chi-square test for association was run on the pairwise categorical variables (arm assignment, whether the participant had had VR therapy, age group, gender, dementia diagnosis, and cognitive level). There was no statistically significant association found between any pairs in any distribution method (*P*>.05). Owing to the small sample of available data for dementia diagnosis, the test was also run with a consolidated list (Alzheimer dementia vs other types of dementia), and no association was found (*P*>.05).

Results from the primary outcomes are described based on the ITT analysis method (n=69 total participants; 34/69, 49% in the VR arm and 35/69, 51% in the control arm). This more conservative method was chosen to minimize bias and provide a closer representation of the effectiveness of VR therapy in clinical practice [68]. The secondary outcomes were evaluated only for patients in the VR arm who received the intervention (29/34, 85%) and are reported per session (n=47 sessions) unless otherwise indicated.

### Primary Outcome Measures

#### BPSD Outcome Measure

Arm assignment was found to have a statistically significant effect on the aggression cluster of BPSDs (Mood median test *P*=.01). Specifically, when comparing the before and after differences (overall median difference=0), participants in the VR arm had a significant decrease in physically aggressive behaviors and loud vocalizations compared with participants in the control arm (Mood median test *P*=.01; Table 2). The Mood median test revealed no statistically significant differences in the NPI-10–like cluster (*P*=.28), wandering cluster (*P*=.70), number of disruptions (*P*=.66), or number of medications refused (*P*=.54).

**Table 2:** Distribution of difference scores (mean number of aggressive events recorded in the electronic medical record after minus before treatment).

Difference^a^ in the mean number of aggressive events	VR^b^ arm (n=34), n (%)	Control arm (n=35), n (%)
More than overall median^c^	4 (12)	13 (37)
Equal to the overall median	19 (56)	17 (49)
Less than the overall median^c^	11 (32)	5 (14)

^a^Difference=after minus before treatment. Difference scores of less than the overall median indicate a relative decrease in aggressive events after virtual reality (VR) therapy. The VR arm had significantly fewer aggressive behaviors after treatment compared with the control arm (mood median test *P*=.01).

^b^VR: virtual reality.

^c^Overall median difference score=0.

#### Falls

Falls were found to have no significant dependence on arm assignment or any other associated factors (Mood median test *P*=.59).

#### Length of Hospital Stay

A Box-Cox logarithmic transformation was conducted on LoS to allow for regression requirements, and a linear regression was run for logarithmic LoS. The model satisfied the linearity independence, equal variance, and normality condition and the low variance inflation factor condition. The regression model showed an overall low explanation for the variance (adjusted *R*^2^=11.5%). LoS showed no statistically significant dependence on arm assignment (*P*=.94).

#### QoL Outcome Measure

Regression analysis found no statistically significant dependence of arm assignment on QoL scores (QUALID instrument) or on any other factor. The regression model satisfied the linearity independence, equal variance, and normality condition and the low variance inflation factor condition but showed no explanation for the variance (adjusted *R*^2^=0%).

Responses from 61% (42/69) of the participants (22/34, 65% from the VR arm and 20/35, 57% from the control arm) were included in the sentiment analysis of the semistructured QoL interview after excluding records in which insufficient data were collected to calculate a difference score (ie, cases with missing data because of excluded responses or in which the semistructured interview was not completed at least once in the before and after periods). When comparing the difference scores for the VR and control arms, the results from the Mood median test approached statistical significance (*P*=.07). Overall, participants in the VR arm had higher difference scores, meaning that, after treatment, they expressed relatively more positive and less negative sentiments when asked questions related to their current well-being and QoL (Table 3).

**Table 3:** Change in overall sentiment during quality of life (QoL) interview after minus before treatment—distribution of difference scores.

Difference in sentiment score^a^	VR^b^ arm (n=34), n (%)	Control arm (n=35), n (%)
Positive	8 (24)	3 (9)
No change	8 (24)	5 (14)
Negative	6 (18)	12 (34)
Unable to assess	12 (35)	15 (43)

^a^Difference=after minus before treatment, where overall positive, neutral, and negative sentiment during the QoL interview were scored as 1, 0, and –1, respectively. Positive difference scores indicate an increase in positive sentiment after treatment.

^b^VR: virtual reality. The VR arm was overall more positive and less negative in their responses compared with the control arm, though this difference was not found to be statistically significant (mood median test *P*=.07).

### Secondary Outcome Measures

Participants completed a mean of 1.6 sessions (SD 0.8) during their stay. Over half (17/29, 59%) of the participants completed 1 session, whereas approximately one-fifth completed 2 (6/29, 21%) and 3 (6/29, 21%) VR therapy sessions.

#### Acceptability of VR

Participants spent an average of 6.8 (SD 6.6; range 0-20) minutes viewing VR films during the sessions. Approximately half (15/29, 52%) of the participants took part in at least one VR session that lasted >5 minutes, whereas 38% (11/29) completed only shorter sessions lasting 1 to 5 minutes. A minority of the participants (3/29, 10%) took part in only a very short VR session or sessions lasting <1 minute, including one individual who opted to listen to music through headphones for 20 minutes instead of using VR because of blindness. For participants who completed more than one session (12/29, 41%), the mean duration of the first VR therapy session was 6.4 (SD 6.0; range 0-20) minutes, and the mean duration of subsequent sessions was 6.3 (SD 6.8; range 0-20) minutes. For participants who completed multiple sessions, the mean difference between the patients’ shortest and longest session was 4.9 (SD 5.7; range 0-16) minutes.

Sessions that ended early were most often because of the participant’s choice (ie, the patient removed the headset with no distress). The reasons for stopping treatment before the end of each 20-minute session are summarized in Table 4.

In some sessions (5/47, 11%), participants showed reluctance or apprehension or anxiety regarding trying VR. For example, one participant initially pushed the headset away, and another one held the HMD throughout the session and appeared reluctant to let go. When present in these cases, informal caregivers (family members) appeared to be helpful in encouraging participation. Additional measures of acceptability (audio and visual stimulus quality, VR HMD comfort, and issues encountered during the sessions) are presented in Table 5. Although most participants indicated that the stimulus quality and headset comfort were adequate, the concern most commonly reported by them was discomfort with the weight of the headset. Observed session issues were most often because of delays related to the acute care setting and unrelated to the VR device (eg, nurses completing a stability check).

**Table 4:** Reasons for stopping virtual reality (VR) therapy sessions (29 participants; n=47 sessions).

		VR sessions, n (%)
Full VR therapy session (20 min)		8 (17)
**Stopped early (<20 min)**		39 (83)
	Participant’s choice (no distress)	31 (66)
	Participant appeared scared, confused, or dizzy (no distress)	3 (6)
	Headset fit related	2 (4)
	Participant choice (low interest)	1 (2)
	Participant experiencing side effects (eye discomfort)	1 (2)
	Participant had a test or treatment	1 (2)

**Table 5:** Acceptability of virtual reality (VR) therapy (29 participants; n=47 sessions).

						VR sessions, n (%)
**Participant responses to postsession questions**						
	**Stimulus quality**					
		Sound was clear			35 (74)	
		Image was in focus			33 (70)	
		**The VR HMD^a^**				
			Was comfortable	35 (74)		
			Was heavy	11 (23)		
			Caused feelings of pressure on the nose or face	6 (13)		
			Felt tight	2 (4)		
			Was uncomfortable	2 (4)		
			Caused eye discomfort (too bright or watery eyes)	2 (4)		
**Observed session issues**						
	Challenges with HMD fit (difficulty adjusting the straps, following instructions [language barrier], and fitting glasses under the HMD)					5 (11)
	Hardware or software technical difficulties (video on pause or headset and controller out of sync)					2 (4)
	Delays unrelated to VR device (locating a nurse for a clinical stability check, patient leaving to use the washroom, patient eating lunch, needing to set up a language line call, confirming assent or dissent procedures, or patient anxious upon RC^b^ arrival)					8 (17)

^a^HMD: head-mounted display.

^b^RC: research coordinator.

#### Safety

No serious side effects or adverse events related to having received VR therapy were observed or reported during the trial. Patients who experienced a serious adverse event during their hospital stay were withdrawn from the study. In the VR arm, adverse events (n=2) that occurred after the first encounter with the participant included stroke (n=1) and death (n=1). These events were determined to be unrelated to having received VR therapy. In the control arm, adverse events (n=5) that occurred after the first encounter with the participant included transfer to the intensive care unit (n=1), seizures (n=2), and death (n=2).

**Figure 4:**
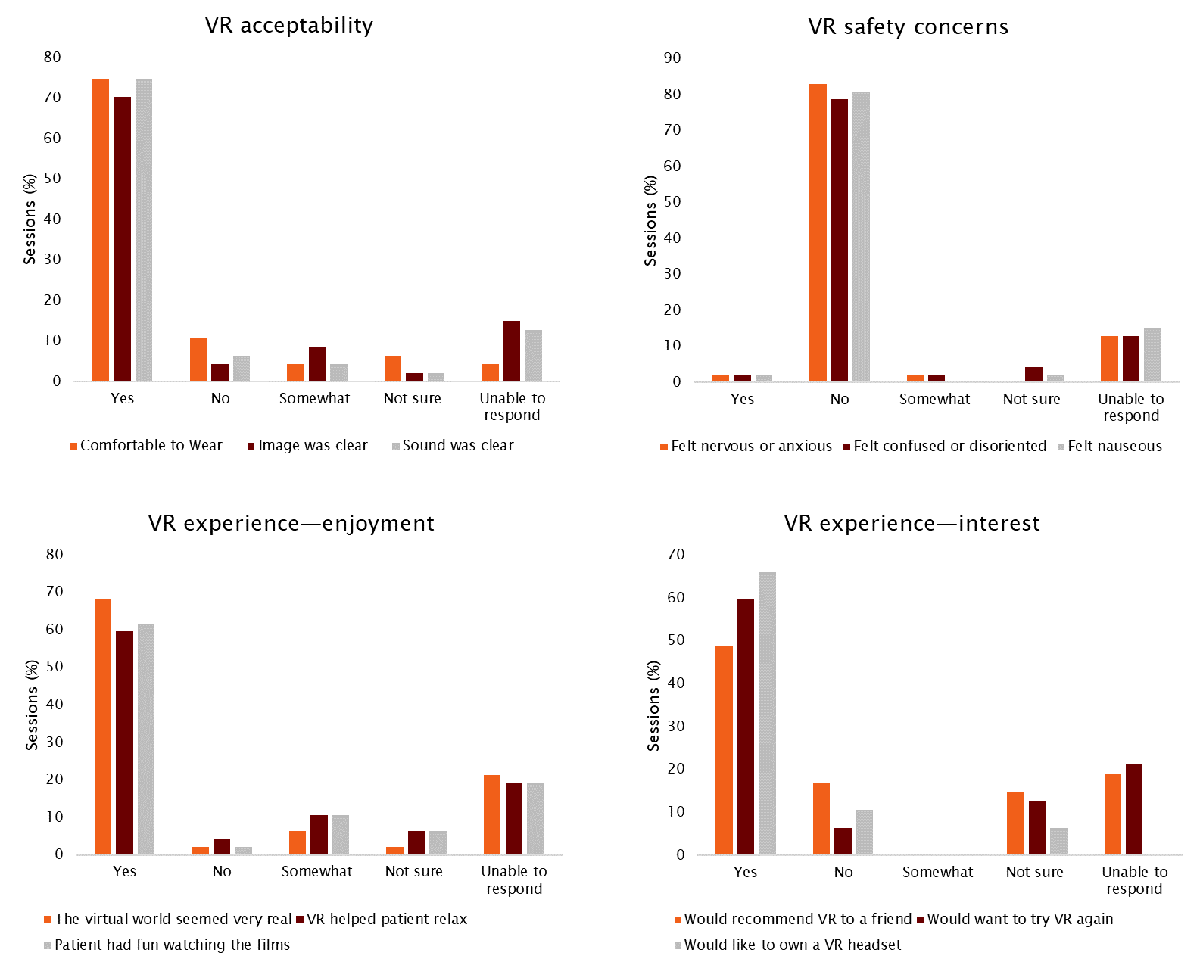
Patient report—virtual reality (VR) acceptability, safety, and experience evaluated from structured postsession feedback interviews (n=47 sessions).

The side effects reported and observed during the VR therapy sessions were mild. During the postsession interview, participants reported feeling at least “a bit” nervous or anxious in 4% (2/47) of the sessions, at least “a bit” confused or disoriented in 4% (2/47) of the sessions, and at least “a bit” nauseous in 2% (1/47) of the sessions (Figure 4). The presence of buzzing sounds from hearing aids could not be evaluated as no participant was wearing a hearing aid during the VR sessions.

#### Participant Experience and Satisfaction With VR Therapy

##### Overview

**Figure 5:**
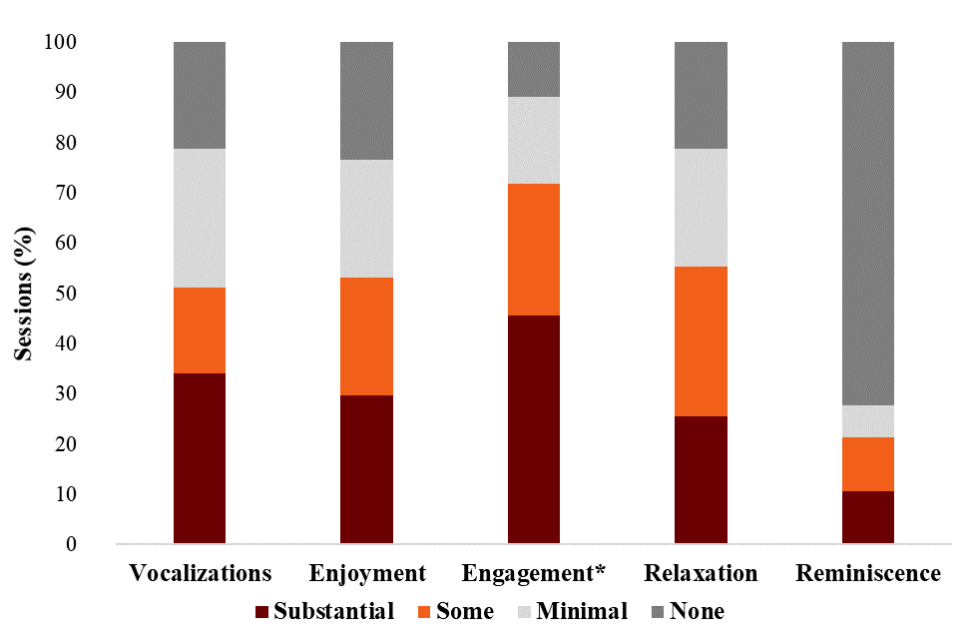
Standardized virtual reality (VR) observation tool results—observed reactions to VR therapy (n=47 sessions; *n=46 sessions).

Structured RC session observations and postsession semistructured interviews with participants indicated that most participants had a positive experience and were satisfied with the VR therapy. Figure 4 summarizes the results of the semistructured interviews. Figure 5 summarizes the results of structured observations using the standardized VR observation tool (vocalizations, enjoyment, relaxation, engagement, and reminiscence; Multimedia Appendix 6).

##### Engagement and Vocalizations

In most sessions, participants demonstrated some or substantial engagement (eg, commenting on or reacting to aspects of the scene) and agreed that the virtual world seemed very real to them. Participants who were more highly engaged interacted with the VR therapy with behaviors such as looking in all directions, pointing at objects or waving at people in the scene, and reacting to changes in scenes with vocalizations such as “Ooh” and “Ah” and moving along with the music. Many participants commented on or described aspects of the scene; for example, “Big trees stretching their hands...near me there is greenery again.” Others who appeared less engaged benefited from prompting and encouragement by the RC or a family caregiver to look around within the scene.

Some participants were more engaged when their caregiver was present, and many appeared happy while chatting with family members present during sessions, often initiating conversations based on content observed in the VR (eg, “Look at this!”). Notably, one participant was observed calling his daughter a pet name that she reported that she had not heard in years.

##### Enjoyment and Reminiscence

Consistent with the RC observations, in most sessions, participants reported that they had fun watching the VR films. Participants who appeared to enjoy their experience were observed laughing and smiling. One participant exclaimed the following while watching the VR:

Wow, I am in the sky! I bless the nurses, they put me in the sky! I have a story to tell when I go home!

One participant began reminiscing about his home country, Tanzania, when he saw water scenes; another described their experience as “Like how we used to watch the water edge [in Sri Lanka].” Another participant said the following:

It’s like in the plane to Jamaica looking down [clapping hands].

Sadness was observed in relatively few sessions (3/47, 6%) and was related to reminiscence in 2 cases.

In the postsession interviews, when asked, “How did you like that,” participants in most of the sessions (32/47, 68%) responded positively and described their experience using words such as “very good,” “very nice,” “lovely,” “beautiful,” and “really enjoyed.” One participant noted that they felt that VR was “good for here in the hospital,” and one described their experience as “Fair enough, a free trip!” Participants in some sessions (6/47, 13%) were more neutral toward their experience or unable to respond to the question (6/47, 13%). Participants in relatively fewer sessions (3/47, 6%) were more negative when describing their experience and cited the weight of the HMD, discomfort, or having a bad day (eg, feeling shaky).

##### Relaxation

Also consistent with RC observations, in over half (28/47, 60%) of the sessions, participants reported that VR therapy helped them relax. In 6% (3/47) of the sessions, participants appeared notably calmer during VR therapy compared with before (ie, stopped restless behaviors such as leg movements or humming and grunting sounds), including one participant who was initially hesitant to try VR.

##### Interest in Trying VR Again

In approximately half (23/47, 49%) of the sessions, participants reported that they would like to own a VR headset, would recommend VR to a friend, and would like to try VR therapy again. Notably, in 13% (6/47) of the sessions, the participants or their caregivers spontaneously mentioned purchasing a VR headset to use at home. For example, one participant said that “I want you to buy for me, Costco maybe,” and another said that “I have to get one of these machines.” In total, 3 caregivers inquired about funding, affordable options, or lighter options for their loved ones with dementia to have access to VR at home.

##### VR Therapy Film Content Preferences

In most cases (37/47, 79%), participants chose to watch multiple mixed scenes during their VR sessions. Participants frequently commented on or reacted to aspects of water or nature scenes (eg, one participant said, “Ah-ha!” each time a water scene came on) or animals and interacted with the music in the videos. Some participants were also observed to fix their gaze in the direction of people in the videos (eg, musicians on a stage and children throwing rocks).

When asked what they wanted to see more of in the VR films, their responses varied. The most frequent suggestions included cultural topics (14/50, 28% of suggestions; eg, art exhibits, varied movie genres, scenic tourist locations, and films with religious locations or themes) and nature scenery (11/50, 22% of suggestions). Films with various animals and types of music were also suggested, as were films incorporating people or personally relevant topics (eg, a participant’s dog). One individual suggested making VR a shared experience (ie, having 2 people involved with 2 HMDs).

## Discussion

### Principal Findings

The aims of this study were to investigate the effects of VR therapy on BPSDs and other related outcomes in people with dementia admitted to an acute care hospital. On the basis of encouraging evidence from recent studies [57], we hypothesized that, by administering VR therapy with natural virtual environments to people with dementia admitted to an acute care hospital, we could help manage BPSDs and improve other process measures, including falls, QoL, and LoS.

In line with our predictions, we found that VR therapy significantly reduced aggressive or violent behavior and loud vocalizations. This is a particularly promising finding as the effective management of aggression has been identified as one of the highest priorities in dementia care [76]. Agitation and its sequelae are common, persistent, and distressing symptoms among people with moderate and severe dementia. In particular, patients with agitation and aggression have difficulty spending time in the hospital [77-79]. Conversely, we found no significant differences between the intervention and control groups for the other BPSDs measured, including the NPI-10–like cluster and wandering. Similarly, we found no significant differences between groups in terms of falls, LoS, or QoL as measured using the QUALID tool.

One possible explanation as to why we observed a positive impact on aggressive behaviors and not on other outcomes relates to VR’s capability to modify known environmental triggers. In this case, patients may have been experiencing pain from acute medical illness; a lack of meaningful stimulation from being in a hospital room; or, similarly, hyperstimulation related to sensory stimuli in the hospital environment. Indeed, VR environments allow individuals to experience a sense of presence and immersion, offering an escape from painful or irritating stimuli [76]. Agitation and aggression are known to respond to environmental interventions that address individuals’ underlying needs, for example, by providing social contact, offering stimulating and relaxing activities, reducing stressful stimuli [80-82], or providing a sense of freedom from confinement [83]. In line with this explanation, we might expect that the short-term effects of VR therapy would be more noticeable for symptoms of agitation provoked by environmental triggers. In contrast, agitation provoked by nonenvironmental factors such as delusional, depressive, and trait anxiety symptoms may require other, more targeted, or extended cognitive and behavioral interventions.

Nevertheless, a critical hindrance to our study and the possible reason for not reaching statistically significant outcomes was the need to cease participant recruitment before reaching the target sample size during the COVID-19 pandemic. Thus, it remains unclear whether this result was due to a lack of statistical power, limited effectiveness of the treatment, or other factors. It is certainly possible that the short intervention period during a typical acute care hospital stay (average LoS in our sample=10.4 days) or number of therapy sessions (mean 1.6 sessions) may have proved insufficient to affect these outcomes in a measurable way. Furthermore, as ours was the first RCT looking at VR therapy for individuals with dementia admitted to an acute care hospital, we were unable to directly compare our findings with those of other similar studies. Nevertheless, we did not observe any clear negative impacts of VR therapy, and we were able to describe factors related to our methodology that may have contributed to the insignificant results.

For measuring BPSDs, analyzing nurses’ notes was determined by the research team to be more feasible than administering the NPI-10 instrument during the pilot that informed this trial [57]. However, it is possible that our positive result for aggression and not for other BPSDs was because aggressive behaviors are more overt and more readily captured in nurses’ notes. Unlike aggressive behaviors, which are externalized, readily visible, and known to respond to environmental interventions within a short period [84], NPI-10–like BPSDs related to trait anxious and depressive symptoms are characterized by internalized features (eg, feelings of dysphoria, euphoria, and delusional beliefs). It is also important to point out that some of these BPSDs, such as anxiety and depression, can be a trigger for aggressive behavior [84,85] and, thus, any improvement may have been captured as a reduction in aggression in our study.

When considering the acceptability, safety, and patient experience with VR therapy, our observational results are overall promising. No serious adverse events occurred that were related to the intervention. Participants reported that audio and visual stimuli were clear in most sessions, and the few reported challenges were due to apprehension or anxiety or mild side effects or discomfort from the HMD. The headset weight was the most commonly reported source of discomfort, which is important to consider given that Oculus Go remains relatively lighter than many HMDs on the market [86]. To reduce discomfort, especially for the frail older population, we recommend considering the HMD weight and adding supports that make the device’s weight distribution more balanced.

Most participants were willing to try VR and were able to tolerate at least a brief session. The average length of exposure was approximately 7 minutes, which is substantially shorter than the median length of treatment protocol of 20 minutes described in other studies using VR to manage BPSDs and promote QoL in people with dementia [65]. This shorter average session time is likely due to participants being acutely ill, although it is unclear whether this constitutes a potential limitation to VR’s effectiveness in people with dementia in acute care as the optimal dosage has not yet been established [65]. Although traditional technologies such as television or music may be easier to administer and better entertain and distract people with dementia in acute care for longer periods, short VR exposures may still be beneficial and arguably easier to administer when compared with longer VR sessions. VR’s unique immersive capabilities make it a particularly powerful tool for eliciting strong positive emotions, including awe, joy, and excitement [87], and brief (6-min) exposure to 360° VR nature videos has been shown to have comparable benefits with those of exposure to the outdoors [88]. Nevertheless, a substantial minority of participants in our study (8/34, 24%) either refused VR or stopped their sessions after <1 minute. We also observed variability in session length within participants; as one participant described, “Today [is] not a good day. Maybe I’d enjoy tomorrow. Just a bit shaky now.” These findings suggest a fluctuating ability to participate and the importance of providing multiple opportunities for patients to try the intervention—and allocating adequate resources to do so, which may pose a challenge in acute care [89,90]. This challenge would not necessarily be unique to VR therapy; although their effectiveness is supported by long-standing clinical experience, nonpharmacological interventions are seldom implemented in practice because of a lack of time and resources [33,91,92] as well as attitudinal barriers [93-96].

In terms of the impact on QoL or well-being, people with dementia in the VR arm consistently provided more positive responses when asked conversational questions about how they were doing, whereas those in the control arm became more negative in their responses after the first study session. Some caregivers also reported a positive impact of VR therapy on their loved ones’ QoL and well-being in general. For example, one caregiver noted the “positive effect” of VR on her mother, and another described VR as “Something to look forward to trying...it explains why he goes out to look at the trees at night*.*” The primary nurse of one patient observed part of a session and reported that she was impressed with the participant’s positive reaction as he had been frequently irritable. Most participants exposed to VR therapy described positive feelings and satisfaction with their experience, with many demonstrating engagement, reminiscence, and relaxation during the sessions. As engaging in relaxation therapy in a natural context may be less practical for this population, especially when in an acute care hospital, we align with other studies in support of providing virtual natural environments to promote well-being and positive experiences [97,98].

In this study, participants were offered the opportunity to choose from a variety of video options and provide feedback on the types of VR content that they would like to see more of. Preferences were varied, and the most frequent suggestions for VR content were cultural topics, followed by nature, animals, and music. Although much remains unknown about the impact of VR content on BPSDs and QoL in people with dementia [51], our findings align with those of other studies that have highlighted the importance of customization of VR contexts in enhancing engagement and allowing for a state of relaxation [99].

### Limitations and Future Directions

This study was subject to several limitations with potential impact on our ability to answer the research questions as well as on the ability to generalize the findings. The first and most critical limitation was the early termination of the study because of the COVID-19 pandemic, with a final recruitment of less than half of the target sample size. Follow-up studies with the sufficient sample size required for statistical power will be necessary to determine whether VR therapy has a significant effect on LoS, falls, QoL, and BPSDs and further establish its effectiveness in reducing aggressive behaviors.

Second, as less than half (12/29, 41%) of the participants completed more than one VR therapy session, it is difficult to draw conclusions regarding treatment effectiveness. Although this limitation is common among similar studies involving VR therapy in people with dementia, it made it more difficult to evaluate the outcomes in general, and it was not possible to measure the impact of treatment dosage or whether there is a wash-out period after repeated VR exposures. As it is not yet understood how the number, frequency, and length of VR therapy sessions correlate with effectiveness [51], it will be important to design future studies that are able to evaluate dose regimens.

Third, we encountered a number of challenges that could be considered inherent to conducting nonpharmacological intervention trials with this population and in this setting. Although the ward and research staff were blinded where possible, neither patients and caregivers nor the RC who administered the VR therapy and collected QoL measures were blinded to treatment allocation, introducing potential for bias. In addition, during recruitment, nearly half (137/300, 45.7%) of the eligible patients screened were unable to participate because they were discharged or deceased by the time their SDM was reached for informed consent. Given this challenge, and considering a typical length of acute care stay of approximately 1 week [100], we recommend using measures that are more sensitive to short-term changes in this setting, particularly for outcomes for which it may take longer to detect any changes as a result of the intervention, such as QoL.

Furthermore, collecting accurate self-report (and proxy-report) data was a challenge, which may have led to insignificant results within our primary outcomes. Through analysis of nurses’ notes, we were able to measure changes in aggression but may have missed capturing less overt outcomes with internalized features (ie, other BPSDs and QoL). Unfortunately, existing self-report measures are difficult to use or inadequate for gathering information from people with dementia [90], and this limited our ability to gather information directly from participants. In our sample up to one-fifth of participants (6/47, 13% to 10/47, 21%) were unable to provide any verbal feedback on their well-being or experience across the various outcome measures that relied on self-report (for which we used supportive communication strategies such as short questions and visual aids). As we anticipated this challenge, we incorporated a standardized VR observation tool to triangulate the self-report findings for the secondary outcomes. Nevertheless, we acknowledge that this methodology presents the possibility of researcher bias, which we took measures to lessen by carefully designing and structuring the tool and providing special training to the researchers involved in data collection. As a future research direction, we recommend designing and validating alternative structured methods that allow for better accessibility for people with dementia [91].

Given these challenges, we suggest focusing on future trials in other care settings (eg, at home, day treatment, or long-term care) to measure effectiveness and continue to refine the intervention with individuals who are less acutely ill. In these cases, changes can be measured over longer periods in a setting in which the individual’s environment is more constant, with more reliable access to family caregivers to help administer VR and provide proxy reports. We are in the process of applying findings from this trial to studies that explore the impact of technological properties on outcomes for both people with dementia and their family caregivers at home [101,102]. Finally, it is worth emphasizing that geriatricians repeatedly report reluctantly administering drugs as they are left with no effective alternatives when dealing with responsive behaviors. An important direction for future studies to explore is whether managing BPSDs using VR therapy might reduce the need for applying restraints or using sedative (antipsychotic and benzodiazepine) medications.

### Conclusions

To the best of our knowledge, this is the first RCT to investigate the impact of VR therapy on BPSDs and well-being in people with dementia in an acute care setting. Although the necessary sample size to observe significance at the desired confidence level was not reached owing to COVID-19 restrictions, we found that immersive VR therapy was effective in reducing aggressive behaviors. In addition, our results suggest nonsignificant but promising trends in terms of VR therapy promoting QoL and add to emerging evidence of VR therapy being a safe and acceptable treatment option for people with dementia. In particular, this is one of the first trials to demonstrate that individuals with moderate to severe dementia and complex medical issues requiring hospitalization can tolerate and enjoy VR for brief periods.

Considering the importance of introducing ethically acceptable and effective techniques to care for people with dementia, rigorous follow-up studies with carefully designed measures are warranted to understand VR therapy’s impact on BPSDs and related outcomes. Given the challenges we encountered in acute care, we recommend first establishing effectiveness and further tailoring the intervention to this population in care settings where individuals are less acutely ill and changes may be more easily detected.

## Appendix

Multimedia Appendix 1 Assent form.

Multimedia Appendix 2 Informed consent form.

Multimedia Appendix 3 Informational study brochure.

Multimedia Appendix 4 Presession checklist.

Multimedia Appendix 5 Modified Quality of Life in Late-Stage Dementia scale.

Multimedia Appendix 6 Standardized virtual reality observation tool.

Multimedia Appendix 7 Baseline demographics table.

Multimedia Appendix 8 Primary outcome data.

Multimedia Appendix 9 Secondary outcome data.

Multimedia Appendix 10 CONSORT-EHEALTH (Consolidated Standards of Reporting Trials of Electronic and Mobile Health Applications and Online Telehealth) checklist [103].

## Abbreviations

BPSD: behavioral and psychological symptom of dementia
CONSORT: Consolidated Standards of Reporting Trials
EMR: electronic medical record
HMD: head-mounted display
ITT: intention to treat
LoS: length of stay
MGH: Michael Garron Hospital
NPI-10: Neuropsychiatric Inventory
QoL: quality of life
QUALID: Quality of Life in Late-Stage Dementia
RC: research coordinator
RCT: randomized controlled trial
SDM: substitute decision-maker
VR: virtual reality

## Data Availability

The data sets supporting the conclusions of this study are included within the paper and its additional files; for example, primary outcome data and secondary outcome data can be found in Multimedia Appendices 8 and 9, respectively.
