# Supplementary material for: Evaluating the Impact of Virtual Reality on the Behavioral and Psychological Symptoms of Dementia and Quality of Life of Inpatients With Dementia in Acute Care: Randomized Controlled Trial (VRCT)
Source: J Med Internet Res. 2024 Jan 30;26:e51758. doi: 10.2196/51758 (PMC10865216; doi:10.2196/51758)
Supplement: Multimedia Appendix 1 [file jmir_v26i1e51758_app1.pdf]

MICHAEL GARRON HOSPITAL
TORONTO EAST HEALTH NETWORK

OPENLAB Proud Member UHN

UHN Toronto General Toronto Western Princess Margaret Toronto Rehab

Patient Information and Assent Form for Research Participation

Study Title: VRx: Randomized Controlled Trial to evaluate the impact of Virtual Reality therapy on quality of life and behavioural and psychological symptoms of individuals with dementia admitted to an acute care hospital.

Sponsor: Centre for Aging and Brain Health Innovation (CABHI)

Principal Investigators: Dr. Howard Abrams (Howard.Abrams@uhn.ca)
Dr. Christopher Smith (Christopher.Smith@tehn.ca)

Co-Investigators: Dr. Lora Appel (Lora.Appel@uhn.ca)
Dr. Jarred Rosenberg (Jarred.Rosenberg@tehn.ca)

Research Contact: Erika Kisonas (Erika.Kisonas@tehn.ca), 416-461-8272 ext. 3279

WHY DO YOU WANT TO TALK TO ME?

We want to tell you about a research study we are doing. We would like to find out more about a tool called Virtual Reality. This form has information to help you choose if you want to be a part of this study.

DO I HAVE TO JOIN THE STUDY?

No, you do not have to join this study. It is your choice.

You can say yes now and change your mind later for any reason. All you have to do is tell us you want to stop. If you stop, your care at the hospital will not change. What people think about you will not change.

WHAT IS VIRTUAL REALITY?

Virtual Reality is a tool that plays images and sounds to make you feel like you are somewhere else. You wear a headset over your eyes and headphones over your ears to use Virtual Reality.

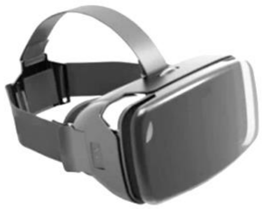

Virtual Reality Headset

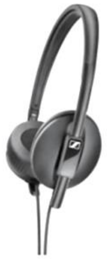

Headphones

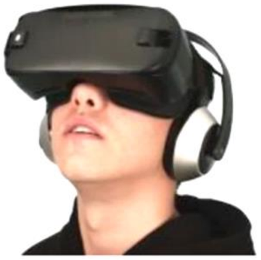

Person Wearing Virtual Reality Headset and Headphones

WHAT WILL I BE ASKED TO DO?

You will put into Group A or Group B. We will tell you if you are in Group A or Group B after you join the study.

A. If you are in Group A, you will answer questions about your mood, watch Virtual Reality films, and answer questions about how Virtual Reality felt. We will ask questions every 1 to 3 days.

OR

B. If you are in Group B, you will answer questions about your mood every 1 to 3 days.

We will visit you in your hospital room. You may stay in bed or be helped into a chair.

WHAT WILL I SEE AND HEAR IN VIRTUAL REALITY?

If you are put into Group A, you will watch Virtual Reality films. These films will make it look and sound like you are in a calming place in nature – like a beach or a forest.

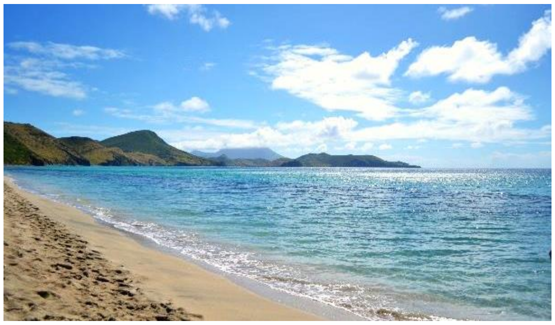

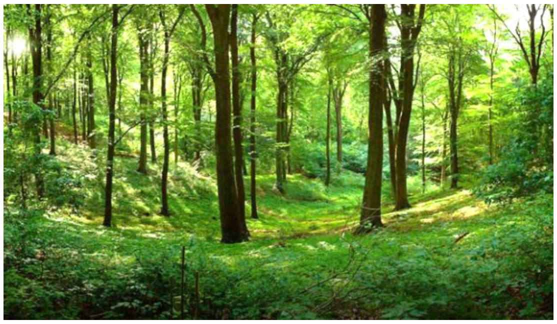

You can watch the films for up to 20 minutes, but you can stop at any time.

WHAT CAN HAPPEN IF I USE VIRTUAL REALITY?

If you watch Virtual Reality films, you may feel sick to your stomach, confused, anxious, or nervous. You may also enjoy watching the Virtual Reality films. They may help you feel calm and relaxed.

EMAIL DISCLAIMER

Please note that the security of email is not guaranteed. Messages may be faked, forwarded or stored by others using the internet. Do not use the e-mail for information you think is sensitive. Do not use e-mail in an emergency since e-mail may be delayed.

Thank you for your time.

Please ask us any questions you have and take your time making a choice.

• 825 Coxwell Avenue, Toronto, ON M4C 3E7 • (416) 461-8272 •
