# Supplementary material for: Evaluating the Impact of Virtual Reality on the Behavioral and Psychological Symptoms of Dementia and Quality of Life of Inpatients With Dementia in Acute Care: Randomized Controlled Trial (VRCT)
Source: J Med Internet Res. 2024 Jan 30;26:e51758. doi: 10.2196/51758 (PMC10865216; doi:10.2196/51758)
Supplement: Multimedia Appendix 2 [file jmir_v26i1e51758_app2.pdf]

PATIENT INFORMATION AND INFORMED CONSENT FORM FOR RESEARCH PARTICIPATION

Study Title: VRx: Randomized Controlled Trial to Evaluate the Impact of Virtual Reality Therapy on Quality of Life and Behavioural and Psychological Symptoms of Individuals with Dementia Admitted to an Acute Hospital.

Investigators:

Principal Investigators: Dr. Howard Abrams (Howard.Abrams@uhn.ca)
Dr. Christopher Smith (Christopher.Smith@tehn.ca)

Co-Investigators: Dr. Lora Appel (Lora.Appel@uhn.ca)
Dr. Jarred Rosenberg (Jarred.Rosenberg@tehn.ca)

EMAIL DISCLAIMER

Please note that the security of email is not guaranteed. Messages may be forged, forwarded or kept indefinitely by others using the internet. Do not use e-mail to discuss information you think is sensitive. Do not use e-mail in an emergency since e-mail may be delayed.

INTRODUCTION

You have expressed an interest in taking part in a research study about Virtual Reality (VR) therapy. This consent form provides you with information to help you make an informed choice. Please read this form carefully and ask any questions you may have.

You may find it helpful to show this form to a friend or family member. All your questions should be answered to your satisfaction before you choose to be a part of this research study. Please take your time in making your decision. A research team member will tell you about the study timelines for making your decision.

You do not need to take part in the study if you don’t want to. You can also leave the study at any time, even if you said you would participate. If you don’t want to participate, or if you want to leave the study, your care at the hospital will not change. If you choose to leave the study, we may still use some of the information that has already been collected, but we will not collect more information.

Eligibility

To be able to participate in the study you must be:

- Over 65 years old.
- Admitted to Michael Garron Hospital.
- Diagnosed with dementia.

To be able to participate in the study you must not have any of the following:

- Alcohol related dementia/ Korsakoff syndrome.
- History of seizures or epilepsy.
- Open wounds on face or head (sutured lacerations exempted).
- Head trauma or stroke leading to your current admission.
- Cervical conditions or injuries that would make it unsafe to use the VR headset.
- Pacemaker
- Admitted to the Intensive Care Unit (ICU) or Adult Mental Health Inpatient Service
- Requires but does not have a contactable Substitute Decision Maker (SDM)
- Has a Public Guardian and Trustee (PGT) as SDM
- Participated in this study during their previous hospitalization and are re-admitted less than 30-days after their previous hospital discharge

Background

Behavioural and Psychological Symptoms of Dementia (BPSD) such as anxiety, restlessness, agitation, aggression, wandering, and depression are common in people with dementia. Current ways to manage these symptoms include medications (neuroleptic /sedating drugs) and applying physical barriers and restraints. Medication may cause heart attacks, falls, tiredness, loss of a self-identity, and faster cognitive decline. Physical restraints may cause pressure sores and infection, worsening anxiety, distress, and acts of physical violence.

Being outside (seeing greenery, hearing sounds from nature) has been shown to make people feel good and reduce depression, anxiety, and physical symptoms of stress.

VR is a new technology that uses a Head Mounted Display (HMD) to produce immersive films that look like real life environments. Viewing these films through the VR display can make you feel as though you are somewhere else. Based on other research and help from experts, we created several VR films that show calming outdoor spaces (peaceful lake, refreshing forest, cheerful playground) made specifically for people with dementia. This may serve as a model for using VR-therapy in hospitals, long term care facilities, and private homes in the future. We expect that VR-therapy will help patients relax,

• 825 Coxwell Avenue, Toronto, ON M4C 3E7 • (416) 461-8272 •

lead to enjoyment, and reduce episodes of BPSD in patients with dementia undergoing treatment at the hospital. We expect that VR-therapy will be safe and comfortable for hospitalized people with dementia to use. We also want to find out if VR-therapy lowers things like length of stay, readmission rates, and falls in hospital.

What is the purpose of the study?
The purpose of this study is to look at the safety and potential benefits of using VR-therapy with patients with dementia admitted to an acute care hospital. If successful, VR-therapy could become a safer, less costly, and more ethical standard of care to help manage BPSD and increase quality of life for people with dementia.

By studying the impact of VR-therapy on patients admitted to an acute care hospital, we are also looking at what resources are needed to introduce a therapy that does not use medication in a hospital setting.

METHODS AND PROCEDURES
Each participant will be randomly assigned to one of two study groups: 1) the group receiving the VR-therapy intervention, or 2) the group receiving the usual standard of care without the VR-therapy intervention. Research team members do not know what group the participant will be a part of before the patient (or person with decision-making authority for the patient) has read, understood, and signed this form.

If you have been assigned to the intervention group, you will watch a series of Virtual Reality (VR) films and answer questions before and after each VR session. If you have been assigned to the usual standard of care group, you will answer some questions but will not receive the VR therapy intervention; you will continue to receive the usual standard of care provided by the hospital.

Virtual Reality is a device that makes your surroundings look and sound like a different place. To experience a VR environment, you wear a headset over your eyes and headphones over your ears (pictured below).

• 825 Coxwell Avenue, Toronto, ON M4C 3E7 • (416) 461-8272 •

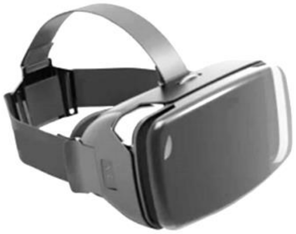

Virtual Reality Headset

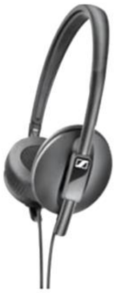

Headphones

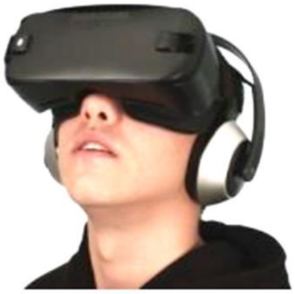

Person Wearing Virtual Reality Headset and Headphones

The VR device will be cleaned according to Michael Garron Hospital's infection control standards before each session. Additionally, a one-time use disposable cover (pictured below) will be secured to the facial cushion during each session.

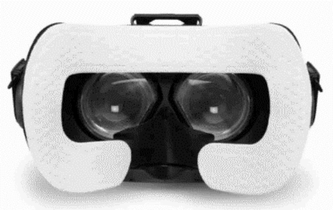

Disposable Facial Cushion Cover

There are no extra physical laboratory tests or medical procedures needed for this study. The intervention is only about watching films using VR equipment and/or answering questions about your experience in the hospital. For the study, the research team will collect data from notes and medical tests conducted as part of your hospital standard of care. No impact on your routine care is expected during the study.

1) **VR-therapy (intervention) group:** If you have been assigned to this group, you will take part in a VR-therapy session every 24-72 hours of your hospital stay. A research team member will make sure your sessions take place at a time that does not conflict with your treatment activities. A research team member will also confirm with a nurse or physician from your ward that you are in stable medical condition to participate in a session that day. The first session will take about 45 minutes and later sessions will take about 25 minutes. At any point in time, before, during, or after the VR-therapy, you can choose to stop being a part of the study. Tell the research team member present that you do not want to continue.

• 825 Coxwell Avenue, Toronto, ON M4C 3E7 • (416) 461-8272 •

Before starting the VR activity, a research team member will ask you some questions about how you feel at that time (e.g. do you feel sad, anxious, depressed, or happy, etc.). This will take about 5 to 10 minutes. You may answer the questions from your hospital bed or a chair in your room.

A research team member will then help you put on the VR device, which is headset where you can watch VR films. The headset looks like ski goggles. A research team member will also help you put on headphones that will allow you to hear the sounds from the VR films. You may watch the VR films from your hospital bed or a chair in your room. You will watch VR films of different lengths (3 to 5 minutes each) and content (such as a lake, forest, playground, etc.). Examples of such nature scenes are shown below. You may watch the VR films for up to 20 minutes.

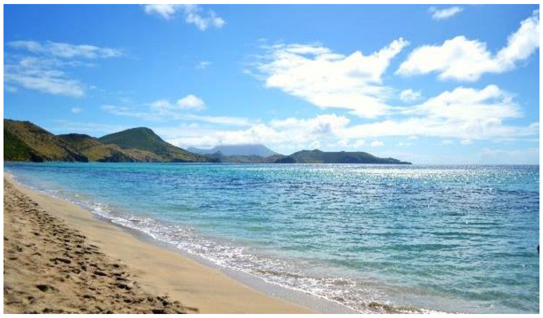

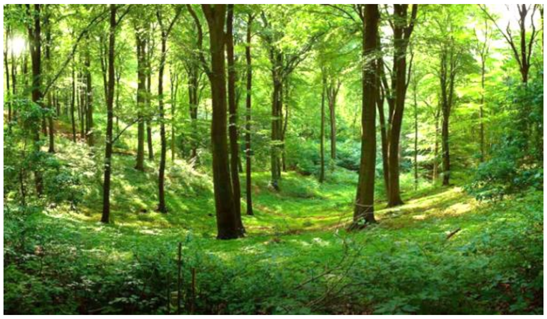

A research team member will watch your reactions during the session and will take notes about your movements, gestures, visual/audio feedback, and other comments you may make. After the VR films, a research team member will ask questions to find out how you felt while watching the films, how you feel after watching the films, and any feedback you have (good or bad) about the films, the VR device, and your experience in general.

2) **Current Standard of Care (no intervention) group:** If you have been assigned to this group, you will take part in a session every 24-72 hours of your hospital stay. A research team member will make sure your sessions take place at a time that does not conflict with your treatment activities. A research team member will also confirm with a nurse or physician from your ward that you are in stable medical condition to participate in a study session that day. The first session will take about 25 minutes and later sessions will take about 10 minutes.

• 825 Coxwell Avenue, Toronto, ON M4C 3E7 • (416) 461-8272 •

You will not watch any VR films if you are placed in this group. A research team member will ask you some questions about how you feel at that time (e.g. do you feel sad, anxious, depressed, or happy, etc.). You may answer the research team member's questions while sitting in your hospital bed or a chair in your room.

At any point in time, before, during, or after the questions, you can choose to stop being a part of the study. Tell a research team member present that you do not want to continue.

CONFIDENTIALITY AND PRIVACY

Your data will be coded with a unique ID number. Identifying information about you will be stored confidentially in locked file cabinets, computer files protected by a password, and a secure web application called Research Electronic Data Capture. Data will be stored for 10 years and then securely destroyed. The only people who will know that you took part in the study are the research team members and the ward staff consulted before each session.

No identifying information about you will be disclosed by Michael Garron Hospital (MGH) to others, University Health Network (UHN) exempted, unless you have said it is ok, except: 1) if needed to protect your rights or welfare (for example, if you are injured and need emergency care); or 2) if required by law. The study results may be written about in a scientific journal or presented at scientific meetings, but your identity will not be shared.

The UHN research team has extensive experience evaluating Virtual Reality applications in Health Care. They are responsible for analyzing the results of the study and guarantee that the information you provide is kept secure.

As part of continuing review of the research, your study records may be accessed on behalf of the Research Ethics Board. A person from the MGH or UHN research ethics team may contact you (if your contact information is available) to ask you questions about the research study and your consent to participate. Your files may be viewed by a member of the MGH or UHN research ethics team for study record auditing purposes. The person viewing your files or contacting you must make sure your information is confidential to the extent permitted by law.

COMPENSATION
You will not have to pay for any of the procedures involved with this study.

You will not be paid for taking part in this study.

The results of this study may lead to the development of marketable treatments or devices that can be sold for profit. Any benefit from commercial products or services will remain with the study sponsor organizations.

POTENTIAL RISKS OF THE STUDY
Some studies warn that unfamiliar technology, such as Virtual Reality, could be confusing to people with cognitive impairment, and may lead to disorientation or agitation. You may also feel motion sickness, or nausea. However, in the past, participants were typically able to use VR without negative results. Participants also often said they would like to watch more VR films in the future.

People who wear hearing aids may sometimes hear high-pitched whistling sounds, buzzes, or static because of interference between the VR device and their hearing aid. If this happens during a VR session, a research team member will ask you to take off your hearing aids when wearing the VR device. A trained member of the research team will be available during the session to address any safety issues or concerns with the participants.

Participants may not feel comfortable sharing personal views during the interview. You can refuse to answer questions or stop taking part in the study at any time.

POTENTIAL BENEFITS FROM THE STUDY
The immediate benefit during the intervention is that you may enjoy watching the VR films. The films may help you feel calm and relaxed. There may be no direct benefit to you from taking part in this study.

The result of this study will help find out if it is safe, feasible and beneficial (such as helping reduce depression, stress, anxiety, wandering and falls, and helping increase feelings of presence and sense of self) for people with dementia to use VR-therapy during their acute care hospital stay.

• 825 Coxwell Avenue, Toronto, ON M4C 3E7 • (416) 461-8272 •

RIGHT TO ASK QUESTIONS AND TO WITHDRAW FROM THE STUDY

You do not need to take part in the study if you don't want to. You can also leave the study at any time, even if you said you would participate. If you don't want to participate, or if you want to leave the study, your care at the hospital will not change.

If you choose to leave the study, we may ask you a few questions about why you are leaving, if you agree to share this information with us. If you leave the study, we may still use some of the information that has already been collected, but we will not collect more information.

The Principal Investigator has the right to withdraw a participant from the study early if they determine the study is not right for them (e.g. participant experiences serious side-effects without any benefits, participant is deliberately providing false information). If the Principal Investigator terminates an individual's participation in the study, the participant/substitute decision maker will be explained the reason why this occurred.

If you have any questions about this study or if you experience a research-related problem or injury, contact:

Principal Investigator: Dr. Christopher Smith
Email: christopher.smith@tehn.ca
Telephone: 416-461-8272 ext. 2570

If you have concerns or questions about your rights as a research participant in the study, feel free to contact:

Chair of the Michael Garron Hospital Research Ethics Board: Dr. Donald Borrett
Telephone: (416) 469-6580 ext. 2967

Chair of the University Health Network Research Ethics Board, Panels A & B (Biomedical): Dr. Morris Sherman
E-mail: dr.morris.sherman@uhn.ca
Telephone: 416-586-4800 ext. 17-8495

University Health Network Research Ethics Board General Inquiries:
Email: reb@uhnresearch.ca
Telephone: 416-581-7849 ext. 18-7849

Participant (or Substitute Decision Maker) Consent to Participation

CONSENT - My signature below indicates:

- I have read the information above.
- I understand the procedures, purpose, risks and benefits of the research study.
- I understand that the results of this study may lead to the development of marketable treatments, devices, or patentable procedures. However, I understand that I will not be entitled to any financial benefits derived from any such commercial developments.
- I have been told that any identifying information will stay confidential.
- I understand I can leave the study at any time.
- I have been given the opportunity to ask questions and all my questions have been answered to my satisfaction.
- I have been given a signed copy of this form.
- I agree to take part in the research as described.

____

Name of Participant (Print) Signature of Participant or SDM Date

VERBAL CONSENT

Was this consent given **verbally** by a Substitute Decision Maker (SDM) who can legally consent on behalf of the participant to take part in this study?
If Yes, please check the relevant box and write below the name of the SDM:

☐ Yes ☐ No

☐ The person providing verbal consent acted as the SDM for the participant during the consent process.

____

Name of SDM (Print) Date of Call Time of Call

SDM Relationship to Participant ____

• 825 Coxwell Avenue, Toronto, ON M4C 3E7 • (416) 461-8272 •

Signature of Person Obtaining Consent

I have personally explained the research to the participant and answered all his/her questions. I believe that s/he understands the information described in this document and freely consents to participate.

Name of Person Obtaining Consent (Print)	Signature	Date

• 825 Coxwell Avenue, Toronto, ON M4C 3E7 • (416) 461-8272 •
