# Supplementary material for: Evaluating the Impact of Virtual Reality on the Behavioral and Psychological Symptoms of Dementia and Quality of Life of Inpatients With Dementia in Acute Care: Randomized Controlled Trial (VRCT)
Source: J Med Internet Res. 2024 Jan 30;26:e51758. doi: 10.2196/51758 (PMC10865216; doi:10.2196/51758)
Supplement: Multimedia Appendix 3 [file jmir_v26i1e51758_app3.pdf]

Study Title: VRx: Randomized Controlled Trial to evaluate the impact of Virtual Reality therapy on quality of life and behavioural and psychological symptoms of individuals with dementia admitted to an acute care hospital.

We want to tell you about a research study we are doing at Michael Garron Hospital. We would like to find out more about a tool called **Virtual Reality.**

This brochure has information to help you choose if you want to take part in this study during your stay in the hospital.

It will not cost you anything to take part in this study.

You do not have to join this study.
It is your choice.

To hear more about the study from our physicians, please visit:

www.PrescribingVR.com/videos

Website: www.PrescribingVR.com

ClinicalTrials.gov Identifier: NCT03941119

Principal Investigators:

Dr. Christopher Smith (Christopher.Smith@tehn.ca)

Dr. Howard Abrams (Howard.Abrams@uhn.ca)

Co-Investigators:

Dr. Lora Appel (Lora.Appel@uhn.ca)

Dr. Jarred Rosenberg (Jarred.Rosenberg@tehn.ca)

Research Contact:

Erika Kisonas (Erika.Kisonas@tehn.ca)

Sponsor:

Centre for Aging and Brain Health Innovation (CABHI)

Michael Garron Hospital
825 Coxwell Ave.
Toronto, ON, M4C 3E7
(416) 461-8272

OpenLab UHN
200 Elizabeth St.
Toronto, ON, M5G 2C4

Virtual Reality Therapy for Patients with Dementia in an Acute Care Hospital: a Randomized Controlled Trial

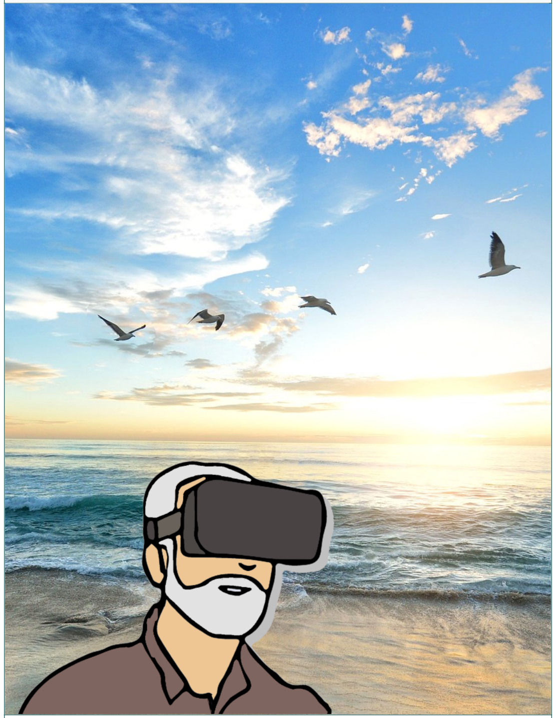

"VRx: Prescribing Virtual Reality" Research Study

MICHAEL GARRON HOSPITAL

WHAT IS VIRTUAL REALITY?

Virtual Reality is a tool that plays images and sounds to make you feel like you are somewhere else. You wear a headset over your eyes and headphones over your ears to use Virtual Reality.

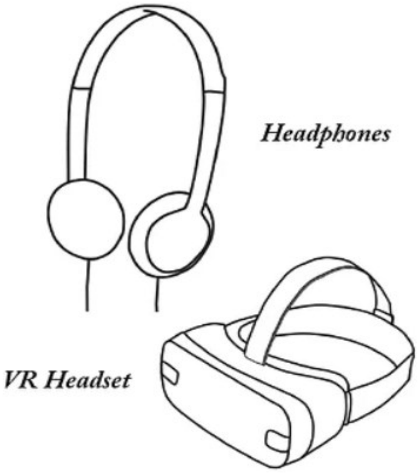

You can watch Virtual Reality films in your bed or in a chair. We will help you put it on and take it off.

WHAT WILL I BE ASKED TO DO?

You will put into Group **A** or Group **B**. We will tell you if you are in Group A or Group B after you join the study.

If you are in Group A, you will answer questions about how you feel in the hospital and watch Virtual Reality films every 1 to 3 days.

OR

If you are in Group B, you will answer questions about how you feel in the hospital every 1 to 3 days.

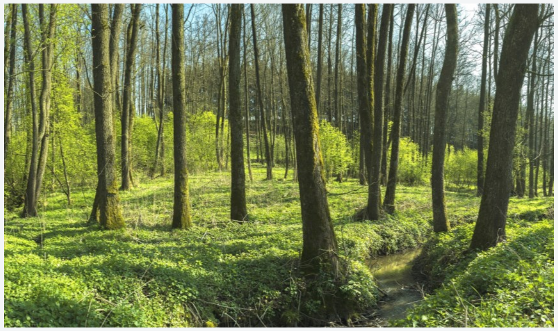

WHAT WILL I SEE AND HEAR?

If you are put into Group A, you will watch Virtual Reality films. These films will make it look and sound like you are in a calming place in nature - like a beach or a forest.

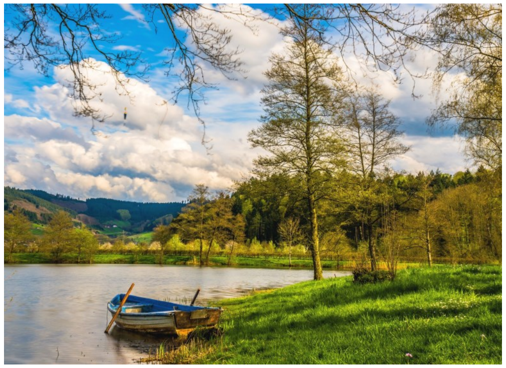

If you are interested, please ask a loved one to contact:

Erika Kisonas, Research Assistant
Email: Erika.Kisonas@tehn.ca
Phone: (416) 461-8272 ext. 3299
