# Supplementary material for: Evaluating the Impact of Virtual Reality on the Behavioral and Psychological Symptoms of Dementia and Quality of Life of Inpatients With Dementia in Acute Care: Randomized Controlled Trial (VRCT)
Source: J Med Internet Res. 2024 Jan 30;26:e51758. doi: 10.2196/51758 (PMC10865216; doi:10.2196/51758)
Supplement: Multimedia Appendix 4 [file jmir_v26i1e51758_app4.pdf]

Nurse Questions

Session #: ____

Session Date and Start Time (time on floor): dd-mmm-yyyy hh:mm

Session Date and End Time (time off floor): dd-mmm-yyyy hh:mm

Name of RC(s) (option to add multiple)

- Erika Kisonas
- Deanna Bartlett
- Jennifer Klein
- Suad Ali
- Eva Appel
- Other: __________

Number of scheduling attempts needed to initiate this session: _____

Check that patient

1) is clinically stable for study session, and
2) does not have any tests/assessments scheduled for the next hour
3) does not refuse to have the session

Proceed only if responses are: **a) Yes, b) No, d) No, and f) No**

Stop if any of the responses to questions listed above are different, respectively

Note: Reponses to questions c) and e) are not a condition to stop the session

**Patient is CLINICALLY STABLE for the study session**	**Response (check one)**	**Assessed by (check all that apply)**
a) Are the patient's vitals stable today?	Yes / No	O Primary nurse<br>O Team lead<br>O Most responsible physician (MRP)<br>O Other staff on ward (specify)<br>________________________
b) Is there any reason why the participant should not undergo a VR session today?	Yes / No	
c) Is there anything else I should know about the participant? If yes, describe:<br>____________________________________	Yes / No	
**Patient's SCHEDULE is CLEAR during the next hour**	**Response (check one)**	**Assessed by (check all that apply)**
d) Does the patient have any tests/assessments scheduled for the next hour?	Yes / No	O Ward Clerk<br>O Other staff on ward (specify)<br>________________________
**Patient DID NOT DISSENT participation in the study Session**	**Response (check one)**	**Assent conducted by (Name of RC)**
e) Was Assent process conducted with the Patient? If No, list reason:	Yes / No	

___________________________________		
f) Did the Patient specifically **DISSENT** (did not agree to) participation in this session? If Yes, provide details: ___________________________________	Yes / No	

Assent conducted by (name of RC): __________

1. If the patient has delirium, checked delirium progression with nurse. If applicable, describe changes in symptoms.
   - O Worsening in hyperactive delirium
   - O No change in hyperactive delirium
   - O Improvement in hyperactive delirium
   - O No change in delirium presentation
   - O N/A (no delirium)
   - O N/A (nurse not available)
   - O Is the patient Alert? (hypoactive delirium)
   - O Worsening in hypoactive delirium
   - O No change in hypoactive delirium
   - O Improvement in hypoactive delirium
   - O Delirium has cleared
   - O Nurse does not know
