# Supplementary material for: Evaluating the Impact of Virtual Reality on the Behavioral and Psychological Symptoms of Dementia and Quality of Life of Inpatients With Dementia in Acute Care: Randomized Controlled Trial (VRCT)
Source: J Med Internet Res. 2024 Jan 30;26:e51758. doi: 10.2196/51758 (PMC10865216; doi:10.2196/51758)
Supplement: Multimedia Appendix 5 [file jmir_v26i1e51758_app5.pdf]

Session Questionnaire

1. How many family members/caregivers are present during this study session?
   (enter a number or Zero if no caregivers are present) ____________________________

2. Specify relationship(s) to patient (check all that apply if one or more caregivers are present)

   - O Daughter
   - O Son
   - O Sister
   - O Brother
   - O Cousin
   - O Uncle
   - O Partner
   - O Wife
   - O Husband
   - O Ex-wife
   - O Ex-husband
   - O Grandson
   - O Granddaughter
   - O Daughter-in-law
   - O Son-in-law
   - O Niece
   - O Nephew
   - O PSW
   - O Friend
   - O Neighbour
   - O Roommate (not related)
   - O Other

Interview (ask participant)

Before starting the study session, ask the participant the following questions.

3. How are you feeling today?
   (If they reply "sometimes" or "some days", ask "What about right now?")

   Open ended reply (if applicable): ____

   Show SMILE scale (if applicable)

   O Very Happy (+2)

   O Somewhat Happy (+1)

   O Neutral (0)

   O Somewhat Sad (-1)

   O Very Sad (-2)

4. Do you remember me?

   O Yes

   O No

   O Not sure

   O N/A

   Comments/open ended reply (if applicable):
   ____

5. How did you sleep?
   Open ended reply (if applicable): ____

   Show SMILE scale (if applicable)

   O Very Happy (+2)

   O Somewhat Happy (+1)

   O Neutral (0)

   O Somewhat Sad (-1)

   O Very Sad (-2)

6. How did you enjoy your meal? (use breakfast, lunch or dinner, depending on the session time)
   Open ended reply (if applicable): ____
   Show SMILE scale (if applicable)

- O Very Happy (+2)
- O Somewhat Happy (+1)
- O Neutral (0)
- O Somewhat Sad (-1)
- O Very Sad (-2)

7. Have you heard of virtual reality before?
   - O Yes
   - O No
   - O Not sure
   - O Other (specify) ______________________________

8. Have you tried a Virtual Reality headset before? (show the VR device to the participant)
   - O Yes
   - O No
   - O Not sure
   - O Other (specify) ______________________________

Observations - Quality of Life (In-Hospital Quality of Life Observational Scale)

Does the patient enjoy interacting or being with others?

-2 -1 0/a 1 2

Does the patient smile?

-2 -1 0/a 1 2

Does the patient appear sad? (sad expression/ voice, tearfulness)

-2 -1 0/a 1 2

Does the patient cry?

-2 -1 0/a 1 2

Does the patient have a facial expression of discomfort? (appears unhappy, in pain, worried, grimaces, furrowed or turned down brow)

-2 -1 0/a 1 2

Does the patient make statements or sounds that suggest discontent, unhappiness, or discomfort? (complains, groans, screams)

-2 -1 0/a 1 2

Does the patient enjoy being touched/ touching?

-2 -1 0/a 1 2

Does the patient appear emotional calm and comfortable?

-2 -1 0/a 1 2

Does the patient appear to be physically uncomfortable? (squirms, frequently changes position)

-2 -1 0/a 1 2

Does the patient enjoy eating? (RC can find this either by asking the patient or the ward staff)

-2 -1 0/a 1 2

Does the patient sleep well? (RC can find this either by asking the patient, the ward staff or from EMR)

-2 -1 0/a 1 2
