# Supplementary material for: Evaluating the Impact of Virtual Reality on the Behavioral and Psychological Symptoms of Dementia and Quality of Life of Inpatients With Dementia in Acute Care: Randomized Controlled Trial (VRCT)
Source: J Med Internet Res. 2024 Jan 30;26:e51758. doi: 10.2196/51758 (PMC10865216; doi:10.2196/51758)
Supplement: Multimedia Appendix 6 [file jmir_v26i1e51758_app6.pdf]

Standardized VR Observation Tool

1. Conversation / Vocalizations - Did the participant initiate conversation or make vocalizations that show interest? For example, ooh’s, ah’s, giggling, or saying “wow”

- Substantial
- Some
- Minimal
- None

2. Perceived enjoyment - Rate the participant’s perceived level of enjoyment during the activity (smiling, laughing, brighter mood/playfulness, sense of humor)

- Substantial
- Some
- Minimal
- None

3. Perceived relaxation - Rate the participant’s perceived level of relaxation during the activity (relaxed mood or muscles, slowed breathing)

- Substantial
- Some
- Minimal
- None

4. Perceived engagement – Did the participant engage with the VR? Did they interact with the 3D environment (looking in many directions, hand movements, seem interested in taking in their surroundings?)

- Substantial
- Some
- Minimal
- None

5. Reminiscence - Did the participant talk about their life experiences (reminiscence) or mention memories meaningful to them during or after VR?

- Substantial
- Some
- Minimal
- None

6. Observations by RC during the VRx experience - (vocalizations, actions, mood, activity level, memories, etc.). Put participant and caregiver quotes in quotation marks (free text)
