# Supplementary material for: Evaluating the Impact of Virtual Reality on the Behavioral and Psychological Symptoms of Dementia and Quality of Life of Inpatients With Dementia in Acute Care: Randomized Controlled Trial (VRCT)
Source: J Med Internet Res. 2024 Jan 30;26:e51758. doi: 10.2196/51758 (PMC10865216; doi:10.2196/51758)
Supplement: Multimedia Appendix 7 [file jmir_v26i1e51758_app7.docx]

**Additional File 7: Baseline Demographic Table**

| Category/Segment (n, %) | Control Arm | VRx Arm | | |  | |
| --- | --- | --- | --- | --- | --- | --- |
|  |  | VRx Arm Total | VRx Arm:  No VRx-Therapy | VRx Arm: With VRx-Therapy | Totals |  |
| Totals | 35, 50.7% | 34, 49.3% | 5, 7.2% | 29, 42.0% | 69, 100.0% |  |
|  |  |  |  |  |  |  |
| Gender |  |  |  |  |  |  |
| - Female | 23, 65.7% | 22, 64.7% | 4, 80.0% | 18, 62.1% | 45, 65.2% |  |
| - Male | 12, 34.3% | 12, 35.3% | 1, 20.0% | 11, 37.9% | 24, 34.8% |  |
|  |  |  |  |  |  |  |
| Age Group |  |  |  |  |  |  |
| - 65-79 | 9, 25.7% | 10, 29.4% | 1, 20.0% | 9, 31.0% | 19, 27.5% |  |
| - 80-89 | 12, 34.3% | 11, 32.4% | 2, 40.0% | 9, 31.0% | 23, 33.3% |  |
| - 90-100 | 14, 40.0% | 13, 38.2% | 2, 40.0% | 11, 37.9% | 27, 39.1% |  |
|  |  |  |  |  |  |  |
| Dementia Type |  |  |  |  |  |  |
| - Dementia | 12, 34.3% | 12, 35.3% | 2, 40.0% | 10, 34.5% | 24, 34.8% |  |
| - Mixed dementia | 13, 37.1% | 9, 26.5% | 0, 0.0% | 9, 31.0% | 22, 31.9% |  |
| - Alzheimer's dementia | 8, 22.9% | 8, 23.5% | 2, 40.0% | 6, 20.7% | 16, 23.2% |  |
| - Vascular dementia | 2, 5.7% | 4, 11.8% | 1, 20.0% | 3, 10.3% | 6, 8.7% |  |
| - Atypical Alzheimer's | 0, 0.0% | 1, 2.9% | 0, 0.0% | 1, 3.4% | 1, 1.4% |  |
|  |  |  |  |  |  |  |
| Cognitive Level |  |  |  |  |  |  |
| - Mild | 11, 31.4% | 7, 20.6% | 1, 20.0% | 6, 20.7% | 18, 26.1% |  |
| - Moderate | 10, 28.6% | 13, 38.2% | 3, 60.0% | 10, 34.5% | 23, 33.3% |  |
| - Severe | 6, 17.1% | 5, 14.7% | 1, 20.0% | 4, 13.8% | 11, 15.9% |  |
| - Unspecified | 8, 22.9% | 9, 26.5% | 0, 0.0% | 9, 31.0% | 17, 24.6% |  |
|  |  |  |  |  |  |  |
| MMSE |  |  |  |  |  |  |
| - Unknown | 22, 62.9% | 23, 67.6% | 1, 20.0% | 22, 75.9% | 45, 65.2% |  |
| - Mild | 7, 20.0% | 4, 11.8% | 3, 60.0% | 1, 3.4% | 11, 15.9% |  |
| - Moderate | 6, 17.1% | 6, 17.6% | 1, 20.0% | 5, 17.2% | 12, 17.4% |  |
| - Severe | 0, 0.0% | 1, 2.9% | 0, 0.0% | 1, 3.4% | 1, 1.4% |  |
|  |  |  |  |  |  |  |
| MoCA |  |  |  |  |  |  |
| - Unknown | 24, 68.6% | 23, 67.6% | 2, 40.0% | 21, 72.4% | 47, 68.1% |  |
| - Mild | 11, 31.4% | 9, 26.5% | 2, 40.0% | 7, 24.1% | 20, 29.0% |  |
| - Moderate | 0, 0.0% | 2, 5.9% | 1, 20.0% | 1, 3.4% | 2, 2.9% |  |

| Charlson Comorbidity | | | | | |
| --- | --- | --- | --- | --- | --- |
| - 0-1 | 4, 11.4% | 4, 11.8% | 0, 0.0% | 4, 13.8% | 8, 11.6% |
| - 2 | 6, 17.1% | 3, 8.8% | 1, 20.0% | 2, 6.9% | 9, 13.0% |
| - 3 | 11, 31.4% | 9, 26.5% | 2, 40.0% | 7, 24.1% | 20, 29.0% |
| - 4 | 8, 22.9% | 9, 26.5% | 2, 40.0% | 7, 24.1% | 17, 24.6% |
| - 5 | 5, 14.3% | 5, 14.7% | 0, 0.0% | 5, 17.2% | 10, 14.5% |
| - 6-9 | 1, 2.9% | 4, 11.8% | 0, 0.0% | 4, 13.8% | 5, 7.2% |
|  |  |  |  |  |  |
| Language |  |  |  |  |  |
| - English | 23, 65.7% | 24, 70.6% | 3, 60.0% | 21, 72.4% | 47, 68.1% |
| - Greek/Macedonian | 4, 11.4% | 6, 17.6% | 2, 40.0% | 4, 13.8% | 10, 14.5% |
| - Other | 8, 22.9% | 4, 11.8% | 0, 0.0% | 4, 13.8% | 12, 17.4% |
|  |  |  |  |  |  |
| Education |  |  |  |  |  |
| - Postgraduate degree | 1, 2.9% | 4, 11.8% | 0, 0.0% | 4, 13.8% | 5, 7.2% |
| - College or university | 8, 22.9% | 7, 20.6% | 2, 40.0% | 5, 17.2% | 15, 21.7% |
| - Graduated from high school | 9, 25.7% | 9, 26.5% | 1, 20.0% | 8, 27.6% | 18, 26.1% |
| - Some high school | 6, 17.1% | 6, 17.6% | 0, 0.0% | 6, 20.7% | 12, 17.4% |
| - Grade 8 or lower | 9, 25.7% | 5, 14.7% | 1, 20.0% | 4, 13.8% | 14, 20.3% |
| - Unknown | 2, 5.7% | 3, 8.8% | 1, 20.0% | 2, 6.9% | 5, 7.2% |
|  |  |  |  |  |  |
| Current Residence |  |  |  |  |  |
| - Assisted living/long term care | 11, 31.4% | 9, 26.5% | 0, 0.0% | 9, 31.0% | 20, 29.0% |
| - Living at home with family member(s) | 9, 25.7% | 10, 29.4% | 1, 20.0% | 9, 31.0% | 19, 27.5% |
| - Living at home alone | 7, 20.0% | 6, 17.6% | 3, 60.0% | 3, 10.3% | 13, 18.8% |
| - Living at home with spouse | 2, 5.7% | 8, 23.5% | 1, 20.0% | 7, 24.1% | 10, 14.5% |
| - Other (specify) | 1, 2.9% | 1, 2.9% | 0, 0.0% | 1, 3.4% | 2, 2.9% |
| - Retirement home/ independent living | 5, 14.3% | 0, 0.0% | 0, 0.0% | 0, 0.0% | 5, 7.2% |
|  |  |  |  |  |  |
| Relationship Status |  |  |  |  |  |
| - Widowed | 21, 60.0% | 13, 38.2% | 3, 60.0% | 10, 34.5% | 34, 49.3% |
| - Married/ partnership | 4, 11.4% | 15, 44.1% | 1, 20.0% | 14, 48.3% | 19, 27.5% |
| - Single | 8, 22.9% | 3, 8.8% | 0, 0.0% | 3, 10.3% | 11, 15.9% |
| - Divorced/separated | 2, 5.7% | 2, 5.9% | 1, 20.0% | 1, 3.4% | 4, 5.8% |
| - Other | 0, 0.0% | 1, 2.9% | 0, 0.0% | 1, 3.4% | 1, 1.4% |
|  |  |  |  |  |  |
| Vision Aids |  |  |  |  |  |
| - None | 13, 37.1% | 13, 38.2% | 2, 40.0% | 11, 37.9% | 26, 37.7% |
| - Reading | 12, 34.3% | 8, 23.5% | 2, 40.0% | 6, 20.7% | 20, 29.0% |
| - Reading & distance | 9, 25.7% | 10, 29.4% | 1, 20.0% | 9, 31.0% | 19, 27.5% |
| - Distance | 1, 2.9% | 3, 8.8% | 0, 0.0% | 3, 10.3% | 4, 5.8% |
|  |  |  |  |  |  |
| Hearing Aids |  |  |  |  |  |
| - None or don't use | 25, 71.4% | 28, 82.4% | 2, 40.0% | 26, 89.7% | 53, 76.8% |
| - Have and use | 10, 28.6% | 6, 17.6% | 3, 60.0% | 3, 10.3% | 16, 23.2% |
|  |  |  |  |  |  |
| Mobility Aids |  |  |  |  |  |
| - Walker | 11, 31.4% | 13, 38.2% | 2, 40.0% | 11, 37.9% | 24, 34.8% |
| - Wheelchair | 7, 20.0% | 9, 26.5% | 2, 40.0% | 7, 24.1% | 16, 23.2% |
| - Cane & Walker | 5, 14.3% | 5, 14.7% | 1, 20.0% | 4, 13.8% | 10, 14.5% |
| - Bedbound | 4, 11.4% | 1, 2.9% | 0, 0.0% | 1, 3.4% | 5, 7.2% |
| - Unknown or Other | 8, 22.9% | 6, 17.6% | 0, 0.0% | 6, 20.7% | 14, 20.3% |
|  |  |  |  |  |  |
| Head Mobility |  |  |  |  |  |
| - Normal | 28, 80.0% | 28, 82.4% | 4, 80.0% | 24, 82.8% | 56, 81.2% |
| - Limited | 7, 20.0% | 6, 17.6% | 1, 20.0% | 5, 17.2% | 13, 18.8% |
|  |  |  |  |  |  |
| Body Mobility (Ability to Sit) | | | | | |
| - Independent | 21, 60.0% | 19, 55.9% | 2, 40.0% | 17, 58.6% | 40, 58.0% |
| - Assisted | 4, 11.4% | 3, 8.8% | 0, 0.0% | 3, 10.3% | 7, 10.1% |
| - Unable | 10, 28.6% | 12, 35.3% | 3, 60.0% | 9, 31.0% | 22, 31.9% |
