# Supplementary material for: Evaluating the Impact of Virtual Reality on the Behavioral and Psychological Symptoms of Dementia and Quality of Life of Inpatients With Dementia in Acute Care: Randomized Controlled Trial (VRCT)
Source: J Med Internet Res. 2024 Jan 30;26:e51758. doi: 10.2196/51758 (PMC10865216; doi:10.2196/51758)
Supplement: Multimedia Appendix 10 [file jmir_v26i1e51758_app10.pdf]

CONSORT-EHEALTH (V 1.6.1) - Submission/Publication Form

The CONSORT-EHEALTH checklist is intended for authors of randomized trials evaluating web-based and Internet-based applications/interventions, including mobile interventions, electronic games (incl multiplayer games), social media, certain telehealth applications, and other interactive and/or networked electronic applications. Some of the items (e.g. all subitems under item 5 - description of the intervention) may also be applicable for other study designs.

The goal of the CONSORT EHEALTH checklist and guideline is to be
a) a guide for reporting for authors of RCTs,
b) to form a basis for appraisal of an ehealth trial (in terms of validity)

CONSORT-EHEALTH items/subitems are MANDATORY reporting items for studies published in the Journal of Medical Internet Research and other journals / scientific societies endorsing the checklist.

Items numbered 1., 2., 3., 4a., 4b etc are original CONSORT or CONSORT-NPT (non-pharmacologic treatment) items.
Items with Roman numerals (i., ii, iii, iv etc.) are CONSORT-EHEALTH extensions/clarifications.

As the CONSORT-EHEALTH checklist is still considered in a formative stage, we would ask that you also RATE ON A SCALE OF 1-5 how important/useful you feel each item is FOR THE PURPOSE OF THE CHECKLIST and reporting guideline (optional).

Mandatory reporting items are marked with a red *.
In the textboxes, either copy & paste the relevant sections from your manuscript into this form - please include any quotes from your manuscript in QUOTATION MARKS,
or answer directly by providing additional information not in the manuscript, or elaborating on why the item was not relevant for this study.

YOUR ANSWERS WILL BE PUBLISHED AS A SUPPLEMENTARY FILE TO YOUR PUBLICATION IN JMIR AND ARE CONSIDERED PART OF YOUR PUBLICATION (IF ACCEPTED).
Please fill in these questions diligently. Information will not be copyedited, so please use proper spelling and grammar, use correct capitalization, and avoid abbreviations.

DO NOT FORGET TO SAVE AS PDF _AND_ CLICK THE SUBMIT BUTTON SO YOUR ANSWERS ARE IN OUR DATABASE !!!

Citation Suggestion (if you append the pdf as Appendix we suggest to cite this paper in the caption):
Eysenbach G, CONSORT-EHEALTH Group
CONSORT-EHEALTH: Improving and Standardizing Evaluation Reports of Web-based and Mobile Health Interventions
J Med Internet Res 2011;13(4):e126

URL: http://www.jmir.org/2011/4/e126/
doi: 10.2196/jmir.1923
PMID: 22209829

madwong6222@gmail.com Switch accounts

Not shared

Draft saved

* Indicates required question

Your name *

First Last

Lora Appel

Primary Affiliation (short), City, Country *

University of Toronto, Toronto, Canada

York University, Toronto, Canada

Your e-mail address *

abc@gmail.com

lappel16@yorku.ca

Title of your manuscript *

Provide the (draft) title of your manuscript.

Evaluating the Impact of Virtual Reality on Behavioural and Psychological Symptoms and Quality of Life of Acute Care Inpatients with Dementia: A Randomized Controlled Trial (VRCT)

Name of your App/Software/Intervention *

If there is a short and a long/alternate name, write the short name first and add the long name in brackets.

Immersive virtual reality (360-degree videos) v

Evaluated Version (if any)

e.g. "V1", "Release 2017-03-01", "Version 2.0.27913"

Not applicable

Language(s) *

What language is the intervention/app in? If multiple languages are available, separate by comma (e.g. "English, French")

English

URL of your Intervention Website or App

e.g. a direct link to the mobile app on app in appstore (itunes, Google Play), or URL of the website. If the intervention is a DVD or hardware, you can also link to an Amazon page.

https://www.prescribingvr.com/vrct

URL of an image/screenshot (optional)

Your answer

Accessibility *

Can an enduser access the intervention presently?

- ○ access is free and open
- ○ access only for special usergroups, not open
- ○ access is open to everyone, but requires payment/subscription/in-app purchases
- ○ app/intervention no longer accessible
- ◉ Other: Future iterations of the intervention described in the present study are

Primary Medical Indication/Disease/Condition *

e.g. "Stress", "Diabetes", or define the target group in brackets after the condition, e.g. "Autism (Parents of children with)", "Alzheimers (Informal Caregivers of)"

Dementia

Primary Outcomes measured in trial *

comma-separated list of primary outcomes reported in the trial

Behavioural and psychological symptoms of d

Secondary/other outcomes

Are there any other outcomes the intervention is expected to affect?

Your answer

Recommended "Dose" *

What do the instructions for users say on how often the app should be used?

- ○ Approximately Daily
- ○ Approximately Weekly
- ○ Approximately Monthly
- ○ Approximately Yearly
- ○ "as needed"
- ◉ Other: The intervention was administered every 24-72 hours, with virtual real

Approx. Percentage of Users (starters) still using the app as recommended after 3 months *

- ◉ unknown / not evaluated
- ○ 0-10%
- ○ 11-20%
- ○ 21-30%
- ○ 31-40%
- ○ 41-50%
- ○ 51-60%
- ○ 61-70%
- ○ 71%-80%
- ○ 81-90%
- ○ 91-100%
- ○ Other:

Overall, was the app/intervention effective? *

- ( ) yes: all primary outcomes were significantly better in intervention group vs control
- (•) partly: SOME primary outcomes were significantly better in intervention group vs control
- ( ) no statistically significant difference between control and intervention
- ( ) potentially harmful: control was significantly better than intervention in one or more outcomes
- ( ) inconclusive: more research is needed
- ( ) Other:

Article Preparation Status/Stage *

At which stage in your article preparation are you currently (at the time you fill in this form)

- ( ) not submitted yet - in early draft status
- ( ) not submitted yet - in late draft status, just before submission
- ( ) submitted to a journal but not reviewed yet
- (•) submitted to a journal and after receiving initial reviewer comments
- ( ) submitted to a journal and accepted, but not published yet
- ( ) published
- ( ) Other:

Journal *

If you already know where you will submit this paper (or if it is already submitted), please provide the journal name (if it is not JMIR, provide the journal name under "other")

- ( ) not submitted yet / unclear where I will submit this
- (•) Journal of Medical Internet Research (JMIR)
- ( ) JMIR mHealth and UHealth
- ( ) JMIR Serious Games
- ( ) JMIR Mental Health
- ( ) JMIR Public Health
- ( ) JMIR Formative Research
- ( ) Other JMIR sister journal
- ( ) Other:

Is this a full powered effectiveness trial or a pilot/feasibility trial? *

- ( ) Pilot/feasibility
- (•) Fully powered

Manuscript tracking number *

If this is a JMIR submission, please provide the manuscript tracking number under "other" (The ms tracking number can be found in the submission acknowledgement email, or when you login as author in JMIR. If the paper is already published in JMIR, then the ms tracking number is the four-digit number at the end of the DOI, to be found at the bottom of each published article in JMIR)

- ( ) no ms number (yet) / not (yet) submitted to / published in JMIR
- (•) Other: JMIR ms#51758

TITLE AND ABSTRACT

1a) TITLE: Identification as a randomized trial in the title

1a) Does your paper address CONSORT item 1a? *

I.e does the title contain the phrase "Randomized Controlled Trial"? (if not, explain the reason under "other")

- (•) yes
- ( ) Other:

1a-i) Identify the mode of delivery in the title

Identify the mode of delivery. Preferably use “web-based” and/or “mobile” and/or “electronic game” in the title. Avoid ambiguous terms like “online”, “virtual”, “interactive”. Use “Internet-based” only if Intervention includes non-web-based Internet components (e.g. email), use “computer-based” or “electronic” only if offline products are used. Use “virtual” only in the context of “virtual reality” (3-D worlds). Use “online” only in the context of “online support groups”. Complement or substitute product names with broader terms for the class of products (such as “mobile” or “smart phone” instead of “iphone”), especially if the application runs on different platforms.

	1	2	3	4	5	
subitem not at all important	( )	( )	(•)	( )	( )	essential

Clear selection

Does your paper address subitem 1a-i? *

Copy and paste relevant sections from manuscript title (include quotes in quotation marks "like this" to indicate direct quotes from your manuscript), or elaborate on this item by providing additional information not in the ms, or briefly explain why the item is not applicable/relevant for your study

“Evaluating the Impact of Virtual Reality”

1a-ii) Non-web-based components or important co-interventions in title

Mention non-web-based components or important co-interventions in title, if any (e.g., “with telephone support”).

	1	2	3	4	5	
subitem not at all important	○	●	○	○	○	essential

Clear selection

Does your paper address subitem 1a-ii?

Copy and paste relevant sections from manuscript title (include quotes in quotation marks "like this" to indicate direct quotes from your manuscript), or elaborate on this item by providing additional information not in the ms, or briefly explain why the item is not applicable/relevant for your study

Our study did not include any co-interventions.

1a-iii) Primary condition or target group in the title

Mention primary condition or target group in the title, if any (e.g., “for children with Type I Diabetes”) Example: A Web-based and Mobile Intervention with Telephone Support for Children with Type I Diabetes: Randomized Controlled Trial

	1	2	3	4	5	
subitem not at all important	○	○	○	○	●	essential

Clear selection

Does your paper address subitem 1a-iii? *

Copy and paste relevant sections from manuscript title (include quotes in quotation marks "like this" to indicate direct quotes from your manuscript), or elaborate on this item by providing additional information not in the ms, or briefly explain why the item is not applicable/relevant for your study

“Acute Care Inpatients with Dementia”

1b) ABSTRACT: Structured summary of trial design, methods, results, and conclusions

NPT extension: Description of experimental treatment, comparator, care providers, centers, and blinding status.

1b-i) Key features/functionalities/components of the intervention and comparator in the METHODS section of the ABSTRACT

Mention key features/functionalities/components of the intervention and comparator in the abstract. If possible, also mention theories and principles used for designing the site. Keep in mind the needs of systematic reviewers and indexers by including important synonyms. (Note: Only report in the abstract what the main paper is reporting. If this information is missing from the main body of text, consider adding it)

	1	2	3	4	5	
subitem not at all important	○	○	○	○	◉	essential

Clear selection

Does your paper address subitem 1b-i? *

Copy and paste relevant sections from the manuscript abstract (include quotes in quotation marks "like this" to indicate direct quotes from your manuscript), or elaborate on this item by providing additional information not in the ms, or briefly explain why the item is not applicable/relevant for your study

“Participants in the experimental (VRx) arm (n=35) were visited by a researcher and watched 360-degree VR-films in a head-mounted display for up to 20 minutes, every 1-3 days, while individuals in the control arm (n=34) received standard of care.”

1b-ii) Level of human involvement in the METHODS section of the ABSTRACT

Clarify the level of human involvement in the abstract, e.g., use phrases like “fully automated” vs. “therapist/nurse/care provider/physician-assisted” (mention number and expertise of providers involved, if any). (Note: Only report in the abstract what the main paper is reporting. If this information is missing from the main body of text, consider adding it)

	1	2	3	4	5	
subitem not at all important	○	○	◉	○	○	essential

Clear selection

Does your paper address subitem 1b-ii?

Copy and paste relevant sections from the manuscript abstract (include quotes in quotation marks "like this" to indicate direct quotes from your manuscript), or elaborate on this item by providing additional information not in the ms, or briefly explain why the item is not applicable/relevant for your study

“Participants in the experimental (VRx) arm (n=35) were visited by a researcher and watched 360-degree VR-films in a head-mounted display for up to 20 minutes, every 1-3 days, while individuals in the control arm (n=34) received standard of care.”

1b-iii) Open vs. closed, web-based (self-assessment) vs. face-to-face assessments in the METHODS section of the ABSTRACT

Mention how participants were recruited (online vs. offline), e.g., from an open access website or from a clinic or a closed online user group (closed usergroup trial), and clarify if this was a purely web-based trial, or there were face-to-face components (as part of the intervention or for assessment). Clearly say if outcomes were self-assessed through questionnaires (as common in web-based trials). Note: In traditional offline trials, an open trial (open-label trial) is a type of clinical trial in which both the researchers and participants know which treatment is being administered. To avoid confusion, use “blinded” or “unblinded” to indicated the level of blinding instead of “open”, as “open” in web-based trials usually refers to “open access” (i.e. participants can self-enrol). (Note: Only report in the abstract what the main paper is reporting. If this information is missing from the main body of text, consider adding it)

	1	2	3	4	5	
subitem not at all important	○	○	◉	○	○	essential

Clear selection

Does your paper address subitem 1b-iii?

Copy and paste relevant sections from the manuscript abstract (include quotes in quotation marks "like this" to indicate direct quotes from your manuscript), or elaborate on this item by providing additional information not in the ms, or briefly explain why the item is not applicable/relevant for your study

“Acute care inpatients with dementia.” “Participants in the experimental (VRx) arm (n=35) were visited by a researcher.” “Instances of daily BPSD and falls were collected from nurses’ daily notes. QoL was measured through semi-structured interviews and The Quality of Life in Late-Stage Dementia scale. Structured observations and semi-structured interviews were used to measure treatment feasibility.” “open-label”

1b-iv) RESULTS section in abstract must contain use data

Report number of participants enrolled/assessed in each group, the use/uptake of the intervention (e.g., attrition/adherence metrics, use over time, number of logins etc.), in addition to primary/secondary outcomes. (Note: Only report in the abstract what the main paper is reporting. If this information is missing from the main body of text, consider adding it)

	1	2	3	4	5	
subitem not at all important	○	○	◉	○	○	essential

Clear selection

Does your paper address subitem 1b-iv?

Copy and paste relevant sections from the manuscript abstract (include quotes in quotation marks "like this" to indicate direct quotes from your manuscript), or elaborate on this item by providing additional information not in the ms, or briefly explain why the item is not applicable/relevant for your study

"The average VR-therapy session lasted 6.8 minutes (range 0-20 minutes)"

1b-v) CONCLUSIONS/DISCUSSION in abstract for negative trials

Conclusions/Discussions in abstract for negative trials: Discuss the primary outcome - if the trial is negative (primary outcome not changed), and the intervention was not used, discuss whether negative results are attributable to lack of uptake and discuss reasons. (Note: Only report in the abstract what the main paper is reporting. If this information is missing from the main body of text, consider adding it)

	1	2	3	4	5	
subitem not at all important	○	○	○	○	◉	essential

Clear selection

Does your paper address subitem 1b-v?

Copy and paste relevant sections from the manuscript abstract (include quotes in quotation marks "like this" to indicate direct quotes from your manuscript), or elaborate on this item by providing additional information not in the ms, or briefly explain why the item is not applicable/relevant for your study

“No statistically significant impact of VR-therapy was found for other BPSD (e.g., apathy), falls, LoS, or QoL as measured through the QUALID scale.” "Although the RCT was stopped before reaching the intended sample size due to COVID-19 restrictions, trends in the results are promising. We suggest conducting future trials with larger samples and, in some cases, more sensitive data collection instruments.”

INTRODUCTION

2a) In INTRODUCTION: Scientific background and explanation of rationale

2a-i) Problem and the type of system/solution

Describe the problem and the type of system/solution that is object of the study: intended as stand-alone intervention vs. incorporated in broader health care program? Intended for a particular patient population? Goals of the intervention, e.g., being more cost-effective to other interventions, replace or complement other solutions? (Note: Details about the intervention are provided in “Methods” under 5)

	1	2	3	4	5	
subitem not at all important	○	○	○	○	◉	essential

Clear selection

Does your paper address subitem 2a-i? *

Copy and paste relevant sections from the manuscript (include quotes in quotation marks "like this" to indicate direct quotes from your manuscript), or elaborate on this item by providing additional information not in the ms, or briefly explain why the item is not applicable/relevant for your study

“Current means to manage BPSD in acute care are insufficient and, at times, pose ethical challenges. Pharmacological therapy, often in the form of atypical antipsychotics, remains the most common treatment for BPSD..."
“Exposure to natural stimuli (e.g., view of trees, flowers, outdoor environment) is a non-pharmacological intervention that has been consistently shown to (1) enhance wellbeing and QoL (2) reduce depression, anxiety, agitation/aggression, and the use of analgesics and (3) and decrease hospital LoS for inpatients38-41.”
“However, providing PwD with exposure to nature is challenging in acute care settings.”
“One way in which people who are unable to engage directly in a natural environment can still be exposed to nature is using Virtual Reality (VR) technology.”

2a-ii) Scientific background, rationale: What is known about the (type of) system

Scientific background, rationale: What is known about the (type of) system that is the object of the study (be sure to discuss the use of similar systems for other conditions/diagnoses, if appropiate), motivation for the study, i.e. what are the reasons for and what is the context for this specific study, from which stakeholder viewpoint is the study performed, potential impact of findings [2]. Briefly justify the choice of the comparator.

	1	2	3	4	5	
subitem not at all important	○	○	○	○	◉	essential

Clear selection

Does your paper address subitem 2a-ii? *

Copy and paste relevant sections from the manuscript (include quotes in quotation marks "like this" to indicate direct quotes from your manuscript), or elaborate on this item by providing additional information not in the ms, or briefly explain why the item is not applicable/relevant for your study

"VR interventions for PwD constitute a cost-effective, non-invasive, and ethically acceptable alternative to manage BPSD through redirecting the patient's attention, within a physically safe environment52,53." "To date, these interventions have typically used music, sensory, and mental stimulation with the aim of improving cognitive functions (attention, executive function, visual and verbal memory), psychological symptoms (agitation, depression, anxiety, and apathy)46,54,55 and the retrieval of autobiographical memories52,56,51..." "Our prior work has shown that it is feasible for frail older adults with varying degrees of physical mobility and cognitive impairment to wear a VR HMD and engage with natural VR environments in a community-care setting52,58..."

2b) In INTRODUCTION: Specific objectives or hypotheses

Does your paper address CONSORT subitem 2b? *

Copy and paste relevant sections from the manuscript (include quotes in quotation marks "like this" to indicate direct quotes from your manuscript), or elaborate on this item by providing additional information not in the ms, or briefly explain why the item is not applicable/relevant for your study

"The main objectives of the current RCT were to evaluate the impact of VR-therapy on BPSD and associated patient outcomes during the hospital stay. Our secondary objectives were to report on the acceptability, safety, experience, and satisfaction with VR-therapy for inpatients with dementia. Specifically, we predicted that for seniors with dementia admitted to an acute care hospital:
1) VR-therapy will help manage BPSD (i.e., decrease anxiety, aggression, depression, violent behaviors, and incidents of wandering), and may decrease the number of incidents that require restraints, LoS, and the number of falls.
2) VR-therapy will improve the QoL (operationalized through validated instruments that measure the QoL for PwD).
3) VR-therapy is safe and feasible (in terms of acceptability and patient experience) to administer."

METHODS

3a) Description of trial design (such as parallel, factorial) including allocation ratio

Does your paper address CONSORT subitem 3a? *

Copy and paste relevant sections from the manuscript (include quotes in quotation marks "like this" to indicate direct quotes from your manuscript), or elaborate on this item by providing additional information not in the ms, or briefly explain why the item is not applicable/relevant for your study

“The current study was an open-label randomized controlled trial conducted at MGH, a community teaching hospital located in Toronto, Canada, in collaboration with OpenLab, an innovation center at University Health Network. The clinical trial protocol was registered with ClinicalTrials.gov, ID:NCT03941119.”
“After obtaining informed consent, the participant was randomized with a 1:1 allocation ratio into either the VRx arm or the Control arm.”

3b) Important changes to methods after trial commencement (such as eligibility criteria), with reasons

Does your paper address CONSORT subitem 3b? *

Copy and paste relevant sections from the manuscript (include quotes in quotation marks "like this" to indicate direct quotes from your manuscript), or elaborate on this item by providing additional information not in the ms, or briefly explain why the item is not applicable/relevant for your study

“The study was temporarily stopped under MGH Research Ethics Board (REB) directive on March 26, 2020, when the first wave of COVID-19 peaked in Canada. Subsequently, recruitment was permanently ceased due to the ongoing pandemic and restrictions on research, specifically with this vulnerable population.”

3b-i) Bug fixes, Downtimes, Content Changes

Bug fixes, Downtimes, Content Changes: ehealth systems are often dynamic systems. A description of changes to methods therefore also includes important changes made on the intervention or comparator during the trial (e.g., major bug fixes or changes in the functionality or content) (5-iii) and other “unexpected events” that may have influenced study design such as staff changes, system failures/downtimes, etc. [2].

	1	2	3	4	5	
subitem not at all important	○	○	●	○	○	essential

Clear selection

Does your paper address subitem 3b-i?

Copy and paste relevant sections from the manuscript (include quotes in quotation marks "like this" to indicate direct quotes from your manuscript), or elaborate on this item by providing additional information not in the ms, or briefly explain why the item is not applicable/relevant for your study

Bug fixes, downtimes and content changes were not relevant to the methods of our study as the VR intervention was delivered by a researcher. Session issues (related to various factors including the virtual reality system, and patient/scheduling factors) are described in the results section.

4a) Eligibility criteria for participants

Does your paper address CONSORT subitem 4a? *

Copy and paste relevant sections from the manuscript (include quotes in quotation marks "like this" to indicate direct quotes from your manuscript), or elaborate on this item by providing additional information not in the ms, or briefly explain why the item is not applicable/relevant for your study

“...Inclusion criteria required that participants be 65 years of age or older, have a documented diagnosis of dementia, and be admitted as an inpatient at MGH. Patients were excluded if they had open facial wounds (sutured lacerations exempted), a history of seizures or epilepsy, a pacemaker, head trauma or stroke leading to their current admission, cervical conditions that would make use of a VR headset unsafe, alcohol-related dementia/Korsakoff syndrome, or were admitted to the Intensive Care Unit (ICU) or Adult Mental Health Inpatient Service..."

4a-i) Computer / Internet literacy

Computer / Internet literacy is often an implicit “de facto” eligibility criterion - this should be explicitly clarified.

	1	2	3	4	5	
subitem not at all important	○	○	◉	○	○	essential

Clear selection

Does your paper address subitem 4a-i?

Copy and paste relevant sections from the manuscript (include quotes in quotation marks "like this" to indicate direct quotes from your manuscript), or elaborate on this item by providing additional information not in the ms, or briefly explain why the item is not applicable/relevant for your study

Computer literacy was neither an explicit nor an implicit eligibility criteria in our study. Our methods describe how the, and how the researcher assisted participants with the technology, including putting on and taking off the VR headset.

4a-ii) Open vs. closed, web-based vs. face-to-face assessments:

Open vs. closed, web-based vs. face-to-face assessments: Mention how participants were recruited (online vs. offline), e.g., from an open access website or from a clinic, and clarify if this was a purely web-based trial, or there were face-to-face components (as part of the intervention or for assessment), i.e., to what degree got the study team to know the participant. In online-only trials, clarify if participants were quasi-anonymous and whether having multiple identities was possible or whether technical or logistical measures (e.g., cookies, email confirmation, phone calls) were used to detect/prevent these.

	1	2	3	4	5	
subitem not at all important	○	○	○	◉	○	essential

Clear selection

Does your paper address subitem 4a-ii? *

Copy and paste relevant sections from the manuscript (include quotes in quotation marks "like this" to indicate direct quotes from your manuscript), or elaborate on this item by providing additional information not in the ms, or briefly explain why the item is not applicable/relevant for your study

"During their hospital stay, recruited patients were visited by the RC every 24-72 hours (1-3 days) who documented findings using a validated QoL scale, a standardized observational tool, semi-structured interviews with participants and their caregivers, and qualitative observations. Any episodes of adverse events (such as vertigo, dizziness, nausea, neck pain, headaches) were documented."

4a-iii) Information giving during recruitment

Information given during recruitment. Specify how participants were briefed for recruitment and in the informed consent procedures (e.g., publish the informed consent documentation as appendix, see also item X26), as this information may have an effect on user self-selection, user expectation and may also bias results.

	1	2	3	4	5	
subitem not at all important	○	○	◉	○	○	essential

Clear selection

Does your paper address subitem 4a-iii?

Copy and paste relevant sections from the manuscript (include quotes in quotation marks "like this" to indicate direct quotes from your manuscript), or elaborate on this item by providing additional information not in the ms, or briefly explain why the item is not applicable/relevant for your study

We have appended the Assent Form, Informed Consent Form, and Informational Study Brochure that were provided to participants (Additional Files 1, 2, and 3).

4b) Settings and locations where the data were collected

Does your paper address CONSORT subitem 4b? *

Copy and paste relevant sections from the manuscript (include quotes in quotation marks "like this" to indicate direct quotes from your manuscript), or elaborate on this item by providing additional information not in the ms, or briefly explain why the item is not applicable/relevant for your study

"The current study was an open-label randomized controlled trial conducted at MGH, a community teaching hospital located in Toronto, Canada, in collaboration with OpenLab, an innovation center at University Health Network."

4b-i) Report if outcomes were (self-)assessed through online questionnaires

Clearly report if outcomes were (self-)assessed through online questionnaires (as common in web-based trials) or otherwise.

	1	2	3	4	5	
subitem not at all important	○	○	◉	○	○	essential

Clear selection

Does your paper address subitem 4b-i? *

Copy and paste relevant sections from the manuscript (include quotes in quotation marks "like this" to indicate direct quotes from your manuscript), or elaborate on this item by providing additional information not in the ms, or briefly explain why the item is not applicable/relevant for your study

“Table 1. Evaluation tools and methods by outcome of interest” in the manuscript provides information on how the data was collected. We did not collect any information through online questionnaires.

4b-ii) Report how institutional affiliations are displayed

Report how institutional affiliations are displayed to potential participants [on ehealth media], as affiliations with prestigious hospitals or universities may affect volunteer rates, use, and reactions with regards to an intervention.(Not a required item – describe only if this may bias results)

	1	2	3	4	5	
subitem not at all important	○	◉	○	○	○	essential

Clear selection

Does your paper address subitem 4b-ii?

Copy and paste relevant sections from the manuscript (include quotes in quotation marks "like this" to indicate direct quotes from your manuscript), or elaborate on this item by providing additional information not in the ms, or briefly explain why the item is not applicable/relevant for your study

Not applicable.

5) The interventions for each group with sufficient details to allow replication, including how and when they were actually administered

5-i) Mention names, credential, affiliations of the developers, sponsors, and owners

Mention names, credential, affiliations of the developers, sponsors, and owners [6] (if authors/evaluators are owners or developer of the software, this needs to be declared in a “Conflict of interest” section or mentioned elsewhere in the manuscript).

	1	2	3	4	5	
subitem not at all important	○	○	○	○	◉	essential

Clear selection

Does your paper address subitem 5-i?

Copy and paste relevant sections from the manuscript (include quotes in quotation marks "like this" to indicate direct quotes from your manuscript), or elaborate on this item by providing additional information not in the ms, or briefly explain why the item is not applicable/relevant for your study

We used off-the-shelf hardware (VR headsets) for this study with videos that were filmed/selected by the research team (not a commercial product). These films were loaded into the internal storage of the VR headset and did not require any additional software. There was no conflict of interest as the researchers do not have a relationship with the developers.
“The Oculus Go, a standalone mobile VR HMD system (discontinued on June 23, 2020), was selected based on its relative affordability and portability65 and reduced likelihood of inducing simulator sickness because of its low motion latency...”

5-ii) Describe the history/development process

Describe the history/development process of the application and previous formative evaluations (e.g., focus groups, usability testing), as these will have an impact on adoption/use rates and help with interpreting results.

	1	2	3	4	5	
subitem not at all important	○	○	○	○	◉	essential

Clear selection

Does your paper address subitem 5-ii?

Copy and paste relevant sections from the manuscript (include quotes in quotation marks "like this" to indicate direct quotes from your manuscript), or elaborate on this item by providing additional information not in the ms, or briefly explain why the item is not applicable/relevant for your study

“A library of short 360-degree films was assembled based on previous studies and expert consultation. The VR films were intentionally chosen to be calming in distinctive ways, as supported by the literature around nature visualization and wellbeing...”
“Our prior work has shown that it is feasible for frail older adults with varying degrees of physical mobility and cognitive impairment to wear a VR HMD and engage with natural VR environments in a community-care setting52,58...”

5-iii) Revisions and updating

Revisions and updating. Clearly mention the date and/or version number of the application/intervention (and comparator, if applicable) evaluated, or describe whether the intervention underwent major changes during the evaluation process, or whether the development and/or content was “frozen” during the trial. Describe dynamic components such as news feeds or changing content which may have an impact on the replicability of the intervention (for unexpected events see item 3b).

	1	2	3	4	5	
subitem not at all important	○	○	○	◉	○	essential

Clear selection

Does your paper address subitem 5-iii?

Copy and paste relevant sections from the manuscript (include quotes in quotation marks "like this" to indicate direct quotes from your manuscript), or elaborate on this item by providing additional information not in the ms, or briefly explain why the item is not applicable/relevant for your study

Not applicable as there was no version number.

5-iv) Quality assurance methods

Provide information on quality assurance methods to ensure accuracy and quality of information provided [1], if applicable.

	1	2	3	4	5	
subitem not at all important	○	○	◉	○	○	essential

Clear selection

Does your paper address subitem 5-iv?

Copy and paste relevant sections from the manuscript (include quotes in quotation marks "like this" to indicate direct quotes from your manuscript), or elaborate on this item by providing additional information not in the ms, or briefly explain why the item is not applicable/relevant for your study

Measures taken to ensure integrity of self-report interview, specific to research in an acute care setting and working with people living with dementia: “During the first visit, the RC conducted a baseline demographic survey with the participant and their informed caregiver (if present)..."
Analysis of primary outcomes: “The categorization and analysis were completed by two coders and reviewed by a third, who were blinded to arm assignment. “
Analysis of secondary outcomes: “Responses to open-ended semi-structured interview questions, RC observations, and participant and caregiver comments during the session were analyzed thematically using NVivo qualitative data analysis computer software package by authors EK, SL, and LA74.”

5-v) Ensure replicability by publishing the source code, and/or providing screenshots/screen-capture video, and/or providing flowcharts of the algorithms used

Ensure replicability by publishing the source code, and/or providing screenshots/screen-capture video, and/or providing flowcharts of the algorithms used. Replicability (i.e., other researchers should in principle be able to replicate the study) is a hallmark of scientific reporting.

	1	2	3	4	5	
subitem not at all important	○	○	◉	○	○	essential

Clear selection

Does your paper address subitem 5-v?

Copy and paste relevant sections from the manuscript (include quotes in quotation marks "like this" to indicate direct quotes from your manuscript), or elaborate on this item by providing additional information not in the ms, or briefly explain why the item is not applicable/relevant for your study

Source code was applicable as off-the shelf hardware was used. Figure 2 provides screen captures of nature scenes from two of the 360-degree films shown to patients.

5-vi) Digital preservation

Digital preservation: Provide the URL of the application, but as the intervention is likely to change or disappear over the course of the years; also make sure the intervention is archived (Internet Archive, webcitation.org, and/or publishing the source code or screenshots/videos alongside the article). As pages behind login screens cannot be archived, consider creating demo pages which are accessible without login.

	1	2	3	4	5	
subitem not at all important	○	○	○	○	◉	essential

Clear selection

Does your paper address subitem 5-vi?

Copy and paste relevant sections from the manuscript (include quotes in quotation marks "like this" to indicate direct quotes from your manuscript), or elaborate on this item by providing additional information not in the ms, or briefly explain why the item is not applicable/relevant for your study

Not applicable as no URL to the intervention because it was not an application (videos only). Figure 2 in the manuscript provides screen captures of nature scenes from two of the 360-degree films shown to patients.

5-vii) Access

Access: Describe how participants accessed the application, in what setting/context, if they had to pay (or were paid) or not, whether they had to be a member of specific group. If known, describe how participants obtained “access to the platform and Internet” [1]. To ensure access for editors/reviewers/readers, consider to provide a “backdoor” login account or demo mode for reviewers/readers to explore the application (also important for archiving purposes, see vi).

	1	2	3	4	5	
subitem not at all important	○	○	◉	○	○	essential

Clear selection

Does your paper address subitem 5-vii? *

Copy and paste relevant sections from the manuscript (include quotes in quotation marks "like this" to indicate direct quotes from your manuscript), or elaborate on this item by providing additional information not in the ms, or briefly explain why the item is not applicable/relevant for your study

“The films were accessed during study sessions by the researcher from the VR HMD’s internal storage.”

5-viii) Mode of delivery, features/functionalities/components of the intervention and comparator, and the theoretical framework

Describe mode of delivery, features/functionalities/components of the intervention and comparator, and the theoretical framework [6] used to design them (instructional strategy [1], behaviour change techniques, persuasive features, etc., see e.g., [7, 8] for terminology). This includes an in-depth description of the content (including where it is coming from and who developed it) [1]," whether [and how] it is tailored to individual circumstances and allows users to track their progress and receive feedback" [6]. This also includes a description of communication delivery channels and – if computer-mediated communication is a component – whether communication was synchronous or asynchronous [6]. It also includes information on presentation strategies [1], including page design principles, average amount of text on pages, presence of hyperlinks to other resources, etc. [1].

	1	2	3	4	5	
subitem not at all important	○	○	○	○	●	essential

Clear selection

Does your paper address subitem 5-viii? *

Copy and paste relevant sections from the manuscript (include quotes in quotation marks "like this" to indicate direct quotes from your manuscript), or elaborate on this item by providing additional information not in the ms, or briefly explain why the item is not applicable/relevant for your study

Theoretical framework: "For some time, guidelines for managing BPSD have recommended using person-centered26-28 psychosocial non-pharmacological interventions as a first-line approach29,30, and qualitative studies exploring the in-hospital experiences of PwD have echoed the need for improved ward-based activity services31,32."
"VR interventions for PwD constitute a cost-effective, non-invasive, and ethically acceptable alternative to manage BPSD through redirecting the patient's attention, within a physically safe environment52,53."
"Our prior work has shown that it is feasible for frail older adults with varying degrees of physical mobility and cognitive impairment to wear a VR HMD and engage with natural VR environments in a community-care setting52,58..."
Tailoring to individual: "the content provided a wide range of features that could be collaboratively selected by the participant, caregiver, or health care provider for each individual in the VRx arm."
Computer-mediated communication was not a component of this intervention.

5-ix) Describe use parameters

Describe use parameters (e.g., intended “doses” and optimal timing for use). Clarify what instructions or recommendations were given to the user, e.g., regarding timing, frequency, heaviness of use, if any, or was the intervention used ad libitum.

	1	2	3	4	5	
subitem not at all important	○	○	○	○	●	essential

Clear selection

Does your paper address subitem 5-ix?

Copy and paste relevant sections from the manuscript (include quotes in quotation marks "like this" to indicate direct quotes from your manuscript), or elaborate on this item by providing additional information not in the ms, or briefly explain why the item is not applicable/relevant for your study

“Patients in the VRx arm then participated in a VR-therapy session for up to 20 minutes. Those in the Control arm otherwise followed the standard of care and were not exposed to VR-therapy. This dosage regimen was informed by previous studies where VR was used to promote wellbeing and manage BPSD in PwD61, as well as other non-pharmacological interventions in acute care.”

5-x) Clarify the level of human involvement

Clarify the level of human involvement (care providers or health professionals, also technical assistance) in the e-intervention or as co-intervention (detail number and expertise of professionals involved, if any, as well as “type of assistance offered, the timing and frequency of the support, how it is initiated, and the medium by which the assistance is delivered”. It may be necessary to distinguish between the level of human involvement required for the trial, and the level of human involvement required for a routine application outside of a RCT setting (discuss under item 21 – generalizability).

	1	2	3	4	5	
subitem not at all important	○	○	○	○	●	essential

Clear selection

Does your paper address subitem 5-x?

Copy and paste relevant sections from the manuscript (include quotes in quotation marks "like this" to indicate direct quotes from your manuscript), or elaborate on this item by providing additional information not in the ms, or briefly explain why the item is not applicable/relevant for your study

“For VRx participants, the RC put the VR headset on the participant’s head, assisted with adjusting the straps for a comfortable fit, and then observed while they watched 360-degree VR films for up to 20 minutes...”

5-xi) Report any prompts/reminders used

Report any prompts/reminders used: Clarify if there were prompts (letters, emails, phone calls, SMS) to use the application, what triggered them, frequency etc. It may be necessary to distinguish between the level of prompts/reminders required for the trial, and the level of prompts/reminders for a routine application outside of a RCT setting (discuss under item 21 – generalizability).

	1	2	3	4	5	
subitem not at all important	○	○	○	○	◉	essential

Clear selection

Does your paper address subitem 5-xi? *

Copy and paste relevant sections from the manuscript (include quotes in quotation marks "like this" to indicate direct quotes from your manuscript), or elaborate on this item by providing additional information not in the ms, or briefly explain why the item is not applicable/relevant for your study

"During their hospital stay, recruited patients were visited by the RC every 24-72 hours (1-3 days)...” “Prior to any study session, depending on the participant allocation (VRx or Control), the RC consulted ward staff to ensure the participant was in stable condition for VR-therapy and had no scheduled treatment or tests in the following hour [see Additional File 4 for Pre-Session Checklist]. As per the study geriatrician’s recommendations, if the participant was asleep upon arrival, the RC attempted to gently wake up the patient by saying their name a few times, or returned later in the day if the patient remained asleep.” In-app reminders were not applicable as all study activities were led by the research coordinator.

5-xii) Describe any co-interventions (incl. training/support)

Describe any co-interventions (incl. training/support): Clearly state any interventions that are provided in addition to the targeted eHealth intervention, as ehealth intervention may not be designed as stand-alone intervention. This includes training sessions and support [1]. It may be necessary to distinguish between the level of training required for the trial, and the level of training for a routine application outside of a RCT setting (discuss under item 21 – generalizability.

	1	2	3	4	5	
subitem not at all important	○	○	○	○	◉	essential

Clear selection

Does your paper address subitem 5-xii? *

Copy and paste relevant sections from the manuscript (include quotes in quotation marks "like this" to indicate direct quotes from your manuscript), or elaborate on this item by providing additional information not in the ms, or briefly explain why the item is not applicable/relevant for your study

There were no co-interventions or training provided. We describe how the researcher provided full assistance to participants to use the intervention: “For VRx participants, the RC put the VR headset on the participant’s head, assisted with adjusting the straps for a comfortable fit, and then observed while they watched 360-degree VR films for up to 20 minutes.”

6a) Completely defined pre-specified primary and secondary outcome measures, including how and when they were assessed

Does your paper address CONSORT subitem 6a? *

Copy and paste relevant sections from the manuscript (include quotes in quotation marks "like this" to indicate direct quotes from your manuscript), or elaborate on this item by providing additional information not in the ms, or briefly explain why the item is not applicable/relevant for your study

“Table 1. Evaluation tools and methods by outcome of interest” provides a full summary of primary and secondary outcome measures and how and when they were assessed.

6a-i) Online questionnaires: describe if they were validated for online use and apply CHERRIES items to describe how the questionnaires were designed/deployed

If outcomes were obtained through online questionnaires, describe if they were validated for online use and apply CHERRIES items to describe how the questionnaires were designed/deployed [9].

	1	2	3	4	5	
subitem not at all important	○	○	◉	○	○	essential

Clear selection

Does your paper address subitem 6a-i?

Copy and paste relevant sections from manuscript text

Not applicable, no online questionnaires were used.

6a-ii) Describe whether and how "use" (including intensity of use/dosage) was defined/measured/monitored

Describe whether and how "use" (including intensity of use/dosage) was defined/measured/monitored (logins, logfile analysis, etc.). Use/adoption metrics are important process outcomes that should be reported in any ehealth trial.

	1	2	3	4	5	
subitem not at all important	○	○	○	◉	○	essential

Clear selection

Does your paper address subitem 6a-ii?

Copy and paste relevant sections from manuscript text

Table 1 describes how "length of time viewing VR" was measured through structured observations, at each VR-therapy session.

6a-iii) Describe whether, how, and when qualitative feedback from participants was obtained

Describe whether, how, and when qualitative feedback from participants was obtained (e.g., through emails, feedback forms, interviews, focus groups).

	1	2	3	4	5	
subitem not at all important	○	○	○	○	◉	essential

Clear selection

Does your paper address subitem 6a-iii?

Copy and paste relevant sections from manuscript text

Table 1 describes how qualitative feedback was collected from participants through semi-structured interviews at each study session (secondary objectives - acceptability, safety, and experience). This is further described in the Methods section. “Acceptability was operationalized through total time spent viewing VR, reported and observed aspects of comfort, and adequacy of the stimuli...”
“Safety was operationalized as any adverse events reported in the EMR, as well as any feelings of nervousness/anxiety, confusion/disorientation, or medical device interaction (i.e., buzzing from hearing aids)...”
“Participants’ experiences during and immediately after VR-therapy were assessed at each study session and operationalized through observations of the level of enjoyment, relaxation, engagement, vocalizations, and presence of reminiscence...”

6b) Any changes to trial outcomes after the trial commenced, with reasons

Does your paper address CONSORT subitem 6b? *

Copy and paste relevant sections from the manuscript (include quotes in quotation marks "like this" to indicate direct quotes from your manuscript), or elaborate on this item by providing additional information not in the ms, or briefly explain why the item is not applicable/relevant for your study

There were no changes to trial outcomes after the trial started.

7a) How sample size was determined

NPT: When applicable, details of whether and how the clustering by care provides or centers was addressed

7a-i) Describe whether and how expected attrition was taken into account when calculating the sample size

Describe whether and how expected attrition was taken into account when calculating the sample size.

	1	2	3	4	5	
subitem not at all important	○	○	◉	○	○	essential

Clear selection

Does your paper address subitem 7a-i?

Copy and paste relevant sections from manuscript title (include quotes in quotation marks "like this" to indicate direct quotes from your manuscript), or elaborate on this item by providing additional information not in the ms, or briefly explain why the item is not applicable/relevant for your study

"With no previous experiments from which to obtain expected significant differences or sample standard deviation, we calculated our sample size based on our feasibility study58 that collected pre-post changes in emotional state using a modified version of the State-Trait Anxiety Inventory (STAI) tool. That research captured pre-post STAI results by categories (called “Emotional Modality”)..”

7b) When applicable, explanation of any interim analyses and stopping guidelines

Does your paper address CONSORT subitem 7b? *

Copy and paste relevant sections from the manuscript (include quotes in quotation marks "like this" to indicate direct quotes from your manuscript), or elaborate on this item by providing additional information not in the ms, or briefly explain why the item is not applicable/relevant for your study

Not applicable - no stopping guidelines were included in the protocol and no interim analyses were conducted.

8a) Method used to generate the random allocation sequence

NPT: When applicable, how care providers were allocated to each trial group

Does your paper address CONSORT subitem 8a? *

Copy and paste relevant sections from the manuscript (include quotes in quotation marks "like this" to indicate direct quotes from your manuscript), or elaborate on this item by providing additional information not in the ms, or briefly explain why the item is not applicable/relevant for your study

“After obtaining informed consent, the participant was randomized into either the VRx arm or the Control arm. Random allocation to one of the two study arms was achieved using a digital tool performing Maximally-Tolerated-Imbalance (MTI) Randomization, a method designed to mitigate selection biases, simultaneously lowering allocation predictability and protecting against chronological bias64.”

8b) Type of randomisation; details of any restriction (such as blocking and block size)

Does your paper address CONSORT subitem 8b? *

Copy and paste relevant sections from the manuscript (include quotes in quotation marks "like this" to indicate direct quotes from your manuscript), or elaborate on this item by providing additional information not in the ms, or briefly explain why the item is not applicable/relevant for your study

There were no restrictions on the randomization: “After obtaining informed consent, the participant was randomized into either the VRx arm or the Control arm. Random allocation to one of the two study arms was achieved using a digital tool performing Maximally-Tolerated-Imbalance (MTI) Randomization, a method designed to mitigate selection biases, simultaneously lowering allocation predictability and protecting against chronological bias64.”

9) Mechanism used to implement the random allocation sequence (such as sequentially numbered containers), describing any steps taken to conceal the sequence until interventions were assigned

Does your paper address CONSORT subitem 9? *

Copy and paste relevant sections from the manuscript (include quotes in quotation marks "like this" to indicate direct quotes from your manuscript), or elaborate on this item by providing additional information not in the ms, or briefly explain why the item is not applicable/relevant for your study

“digital tool performing Maximally-Tolerated-Imbalance (MTI) Randomization, a method designed to mitigate selection biases, simultaneously lowering allocation predictability and protecting against chronological bias64.”

10) Who generated the random allocation sequence, who enrolled participants, and who assigned participants to interventions

Does your paper address CONSORT subitem 10? *

Copy and paste relevant sections from the manuscript (include quotes in quotation marks "like this" to indicate direct quotes from your manuscript), or elaborate on this item by providing additional information not in the ms, or briefly explain why the item is not applicable/relevant for your study

We describe how the research team first screened participants, obtained informed consent and assent before enrolling participants, and then generated the random allocation sequence after consent to assign participants to interventions:
“All new patient admissions to the hospitalist/internal medicine department at MGH were screened daily, excluding weekends and statutory holidays, using the hospital’s Electronic Medical Record (EMR) system.”
“If the patient had a documented capacity assessment determining they were able to provide informed consent, the RC approached them in-person to ask about their interest in the study...”
“After obtaining informed consent, the participant was randomized into either the VRx arm or the Control arm.”

11a) If done, who was blinded after assignment to interventions (for example, participants, care providers, those assessing outcomes) and how

NPT: Whether or not administering co-interventions were blinded to group assignment

11a-i) Specify who was blinded, and who wasn’t

Specify who was blinded, and who wasn’t. Usually, in web-based trials it is not possible to blind the participants [1, 3] (this should be clearly acknowledged), but it may be possible to blind outcome assessors, those doing data analysis or those administering co-interventions (if any).

	1	2	3	4	5	
subitem not at all important	○	○	○	○	◉	essential

Clear selection

Does your paper address subitem 11a-i? *

Copy and paste relevant sections from the manuscript (include quotes in quotation marks "like this" to indicate direct quotes from your manuscript), or elaborate on this item by providing additional information not in the ms, or briefly explain why the item is not applicable/relevant for your study

“After randomization, the participants, caregivers, and research personnel were not blind to treatment allocation, given the nature of the intervention. The ward staff were blinded to treatment allocation to the greatest extent possible. Participants and caregivers were asked not to discuss their arm allocation with the ward staff.”

11a-ii) Discuss e.g., whether participants knew which intervention was the “intervention of interest” and which one was the “comparator”

Informed consent procedures (4a-ii) can create biases and certain expectations - discuss e.g., whether participants knew which intervention was the “intervention of interest” and which one was the “comparator”.

	1	2	3	4	5	
subitem not at all important	○	○	○	○	●	essential

Clear selection

Does your paper address subitem 11a-ii?

Copy and paste relevant sections from the manuscript (include quotes in quotation marks "like this" to indicate direct quotes from your manuscript), or elaborate on this item by providing additional information not in the ms, or briefly explain why the item is not applicable/relevant for your study

“The current study was an open-label randomized controlled trial.” There was no comparator intervention. The appended Consent and Assent forms (Additional Files 1 and 2) describe to participants how they will be randomized to receive VR therapy or no intervention (control arm).

11b) If relevant, description of the similarity of interventions

(this item is usually not relevant for ehealth trials as it refers to similarity of a placebo or sham intervention to a active medication/intervention)

Does your paper address CONSORT subitem 11b? *

Copy and paste relevant sections from the manuscript (include quotes in quotation marks "like this" to indicate direct quotes from your manuscript), or elaborate on this item by providing additional information not in the ms, or briefly explain why the item is not applicable/relevant for your study

Not applicable as there was no comparator intervention.“Those in the Control arm otherwise followed the standard of care and were not exposed to VR-therapy.”

12a) Statistical methods used to compare groups for primary and secondary outcomes

NPT: When applicable, details of whether and how the clustering by care providers or centers was addressed

Does your paper address CONSORT subitem 12a? *

Copy and paste relevant sections from the manuscript (include quotes in quotation marks "like this" to indicate direct quotes from your manuscript), or elaborate on this item by providing additional information not in the ms, or briefly explain why the item is not applicable/relevant for your study

“Analysis: Statistical analyses were performed using the MiniTAB statistical package67. The Anderson-Darling test for normality was run for all variables to determine whether to use statistical regression or a nonparametric test. Linear regression was used for outcomes with normally distributed values (Anderson-Darling P>.05), with arm assignment (VRx vs. Control) as the main factor...
Primary Outcome Measures: The primary outcomes in this study pertained to the effects of VR-therapy on behavioral and psychological symptoms of dementia and other critical acute care outcomes. Data were analyzed at a 95% significance level, based on ITT and Per-Protocol analysis methods68-70...
Behavioral and Psychological Symptoms of Dementia: Data on BPSD were collected from nurses’ notes in the EMR that reported daily patient symptoms. Key terms from the nurses’ notes were then categorized and analyzed based on the following predetermined clusters...
Falls: Outcomes concerning falls were operationalized as the frequencies of: 1) additional falls precautions applied, 2) falls with injury, and 3) falls without injury during the hospital stay, as recorded in the EMR.
Length of Stay: Length of acute hospital stay was calculated as the difference in minutes between “Discharge Date/Time” and “Admission Date/Time”, as recorded in the EMR.
Quality of Life: QoL was measured through an adapted version of the QUALID scale73. This adapted scale [see Additional File 5] was administered by the RC at the beginning of each study session...
Secondary Outcome Measures: Through the secondary outcomes, we sought to investigate the safety, acceptability and feasibility of administering VR-therapy for inpatients with dementia in an acute care setting. The secondary outcomes were analyzed by session, and only for patients in the VRx arm who received VR-therapy..."

12a-i) Imputation techniques to deal with attrition / missing values

Imputation techniques to deal with attrition / missing values: Not all participants will use the intervention/comparator as intended and attrition is typically high in ehealth trials. Specify how participants who did not use the application or dropped out from the trial were treated in the statistical analysis (a complete case analysis is strongly discouraged, and simple imputation techniques such as LOCF may also be problematic [4]).

	1	2	3	4	5	
subitem not at all important	○	○	○	◉	○	essential

Clear selection

Does your paper address subitem 12a-i? *

Copy and paste relevant sections from the manuscript (include quotes in quotation marks "like this" to indicate direct quotes from your manuscript), or elaborate on this item by providing additional information not in the ms, or briefly explain why the item is not applicable/relevant for your study

Quality of Life: “Responses from 42 participants (VRx arm n = 22, Control arm n = 20) were included in the sentiment analysis of the semi-structured QoL interview after excluding records where insufficient data were collected to calculate a difference score (i.e., cases with missing data due to excluded responses, or where the semi-structured interview was not completed at least once in the Before and After periods).” “From each of the two arms, four participants were excluded as there was no encounter with the participants to allow for meaningful comparison (i.e., the only interaction occurred on the last day of the hospital stay), leaving n = 69 participants in total, with n = 35 in the Control arm and n = 34 in the experimental VR-therapy (VRx) arm. In the VRx arm, 29/34 participants (85%) completed at least one session of VR-therapy, while 5/34 (15%) refused the VR and completed no VR-therapy sessions. See Figure 3 for a Consort diagram of patient flow based on Intention-to-Treat (ITT).”

12b) Methods for additional analyses, such as subgroup analyses and adjusted analyses

Does your paper address CONSORT subitem 12b? *

Copy and paste relevant sections from the manuscript (include quotes in quotation marks "like this" to indicate direct quotes from your manuscript), or elaborate on this item by providing additional information not in the ms, or briefly explain why the item is not applicable/relevant for your study

Primary outcomes: “To determine whether differences could be attributed to the treatment effect rather than individual characteristics, other factors included in the regression analyses were Had VR (whether or not the participant had VR-therapy sessions), Age Group, Gender, Cognitive Level, and Dementia Type.”
Secondary outcomes: “Observational data from first sessions vs. any subsequent sessions were compared manually to determine whether there were any clear trends that would warrant analysis by participant vs. session. Descriptive statistics revealed no clear patterns of differences that would warrant separate analysis by participant as opposed to by session.”

X26) REB/IRB Approval and Ethical Considerations [recommended as subheading under "Methods"] (not a CONSORT item)

X26-i) Comment on ethics committee approval

	1	2	3	4	5	
subitem not at all important	○	○	○	○	◉	essential

Clear selection

Does your paper address subitem X26-i?

Copy and paste relevant sections from the manuscript (include quotes in quotation marks "like this" to indicate direct quotes from your manuscript), or elaborate on this item by providing additional information not in the ms, or briefly explain why the item is not applicable/relevant for your study

“REB approval for research involving human subjects was received from the Michael Garron Hospital REB, reference number 782-1812-Mis-332 on January 14th, 2019.”

x26-ii) Outline informed consent procedures

Outline informed consent procedures e.g., if consent was obtained offline or online (how? Checkbox, etc.?), and what information was provided (see 4a-ii). See [6] for some items to be included in informed consent documents.

	1	2	3	4	5	
subitem not at all important	○	○	○	◉	○	essential

Clear selection

Does your paper address subitem X26-ii?

Copy and paste relevant sections from the manuscript (include quotes in quotation marks "like this" to indicate direct quotes from your manuscript), or elaborate on this item by providing additional information not in the ms, or briefly explain why the item is not applicable/relevant for your study

“The consent process followed Ontario’s legislation62, and the hospital provided a brochure63 to help patients and families understand the different roles involved in decision-making. Any previously documented capacity assessments were respected. For potential participants with no record of capacity assessment, capacity for providing informed consent was obtained as per the study site protocol..."

X26-iii) Safety and security procedures

Safety and security procedures, incl. privacy considerations, and any steps taken to reduce the likelihood or detection of harm (e.g., education and training, availability of a hotline)

	1	2	3	4	5	
subitem not at all important	○	○	○	◉	○	essential

Clear selection

Does your paper address subitem X26-iii?

Copy and paste relevant sections from the manuscript (include quotes in quotation marks "like this" to indicate direct quotes from your manuscript), or elaborate on this item by providing additional information not in the ms, or briefly explain why the item is not applicable/relevant for your study

Privacy: “Data was anonymized and de-identified at the time of collection (i.e., linked only to study ID).”
Safety: “Prior to any study session, depending on the participant allocation (VRx or Control), the RC consulted ward staff to ensure the participant was in stable condition for VR-therapy and had no scheduled treatment or tests in the following hour [see Additional File 4 for Pre-Session Checklist]..."
The RC was present with the participant for the duration of the session: “For VRx participants, the RC put the VR headset on the participant’s head, assisted with adjusting the straps for a comfortable fit, and then observed while they watched 360-degree VR films for up to 20 minutes.”

RESULTS

13a) For each group, the numbers of participants who were randomly assigned, received intended treatment, and were analysed for the primary outcome

NPT: The number of care providers or centers performing the intervention in each group and the number of patients treated by each care provider in each center

Does your paper address CONSORT subitem 13a? *

Copy and paste relevant sections from the manuscript (include quotes in quotation marks "like this" to indicate direct quotes from your manuscript), or elaborate on this item by providing additional information not in the ms, or briefly explain why the item is not applicable/relevant for your study

“Results from Primary Outcomes are described based on the ITT analysis method (n = 69 participants total; n = 34 in the VRx arm, n = 35 in the Control arm).”

13b) For each group, losses and exclusions after randomisation, together with reasons

Does your paper address CONSORT subitem 13b? (NOTE: Preferably, this is shown in a CONSORT flow diagram) *

Copy and paste relevant sections from the manuscript (include quotes in quotation marks "like this" to indicate direct quotes from your manuscript), or elaborate on this item by providing additional information not in the ms, or briefly explain why the item is not applicable/relevant for your study

This information is shown in a CONSORT flow diagram and described in the results:
“After contacting substitute decision makers (SDM) of eligible participants, and excluding patients who met exclusion criteria (i.e., discharged/deceased), or refused to use the VR equipment, a total of 77 patients with dementia were consented and enrolled in the study. Participants were randomized into the Control arm with no VR-therapy exposure (n = 39) and the Intervention arm receiving VR-therapy once every 1-3 days) (n = 38). From each of the two arms, four participants were excluded as there was no encounter with the participants to allow for meaningful comparison (i.e., the only interaction occurred on the last day of the hospital stay), leaving n = 69 participants in total, with n = 35 in the Control arm and n = 34 in the experimental VR-therapy (VRx) arm.”

13b-i) Attrition diagram

Strongly recommended: An attrition diagram (e.g., proportion of participants still logging in or using the intervention/comparator in each group plotted over time, similar to a survival curve) or other figures or tables demonstrating usage/dose/engagement.

	1	2	3	4	5	
subitem not at all important	○	○	●	○	○	essential

Clear selection

Does your paper address subitem 13b-i?

Copy and paste relevant sections from the manuscript or cite the figure number if applicable (include quotes in quotation marks "like this" to indicate direct quotes from your manuscript), or elaborate on this item by providing additional information not in the ms, or briefly explain why the item is not applicable/relevant for your study

We do not include an attrition diagram, however we do report on reasons for withdrawal prior to receiving treatment: “In the VRx arm, 29/34 participants (85%) completed at least one session of VR-therapy, while 5/34 (15%) refused the VR and completed no VR-therapy sessions.”
As well as usage/participation: “Participants completed a mean of 1.6 sessions during their stay. Over half of participants (17/29, 58.6%) completed one session, while approximately one fifth of participants (6/29 20.7%) completed two and three (6/29, 20.7%) VR-therapy sessions, respectively..."

14a) Dates defining the periods of recruitment and follow-up

Does your paper address CONSORT subitem 14a? *

Copy and paste relevant sections from the manuscript (include quotes in quotation marks "like this" to indicate direct quotes from your manuscript), or elaborate on this item by providing additional information not in the ms, or briefly explain why the item is not applicable/relevant for your study

“Data collection occurred between April 29, 2019 and March 13, 2020. The study was temporarily stopped under MGH Research Ethics Board (REB) directive on March 26, 2020, when the first wave of COVID-19 peaked in Canada. Subsequently, recruitment was permanently ceased due to the ongoing pandemic and restrictions on research, specifically with this vulnerable population.”

14a-i) Indicate if critical “secular events” fell into the study period

Indicate if critical “secular events” fell into the study period, e.g., significant changes in Internet resources available or “changes in computer hardware or Internet delivery resources”

	1	2	3	4	5	
subitem not at all important	○	◉	○	○	○	essential

Clear selection

Does your paper address subitem 14a-i?

Copy and paste relevant sections from the manuscript (include quotes in quotation marks "like this" to indicate direct quotes from your manuscript), or elaborate on this item by providing additional information not in the ms, or briefly explain why the item is not applicable/relevant for your study

Not applicable/did not occur.

14b) Why the trial ended or was stopped (early)

Does your paper address CONSORT subitem 14b? *

Copy and paste relevant sections from the manuscript (include quotes in quotation marks "like this" to indicate direct quotes from your manuscript), or elaborate on this item by providing additional information not in the ms, or briefly explain why the item is not applicable/relevant for your study

“The study was temporarily stopped under MGH Research Ethics Board (REB) directive on March 26, 2020, when the first wave of COVID-19 peaked in Canada. Subsequently, recruitment was permanently ceased due to the ongoing pandemic and restrictions on research, specifically with this vulnerable population.”

15) A table showing baseline demographic and clinical characteristics for each group

NPT: When applicable, a description of care providers (case volume, qualification, expertise, etc.) and centers (volume) in each group

Does your paper address CONSORT subitem 15? *

Copy and paste relevant sections from the manuscript (include quotes in quotation marks "like this" to indicate direct quotes from your manuscript), or elaborate on this item by providing additional information not in the ms, or briefly explain why the item is not applicable/relevant for your study

“Additional File 7 describes the baseline demographics of all study participants (n = 69), as reported in clinical documentation based on three groups: “Control arm”, “VRx arm: With VRx-therapy”, and “VRx arm: No VRx-therapy..."

15-i) Report demographics associated with digital divide issues

In ehealth trials it is particularly important to report demographics associated with digital divide issues, such as age, education, gender, social-economic status, computer/Internet/ehealth literacy of the participants, if known.

	1	2	3	4	5	
subitem not at all important	○	○	○	○	◉	essential

Clear selection

Does your paper address subitem 15-i? *

Copy and paste relevant sections from the manuscript (include quotes in quotation marks "like this" to indicate direct quotes from your manuscript), or elaborate on this item by providing additional information not in the ms, or briefly explain why the item is not applicable/relevant for your study

We report on age, gender, and relevant medical conditions that would impact individuals’ ability to interact with the VR technology (e.g., cognition, hearing, vision, ability to sit independently). We did not measure technology literacy as none was required to participate in the study (full assistance was provided to use VR).
“Additional File 7 describes the baseline demographics of all study participants (n = 69), as reported in clinical documentation based on three groups: “Control arm”, “VRx arm: With VRx-therapy”, and “VRx arm: No VRx-therapy...”

16) For each group, number of participants (denominator) included in each analysis and whether the analysis was by original assigned groups

16-i) Report multiple “denominators” and provide definitions

Report multiple “denominators” and provide definitions: Report N’s (and effect sizes) “across a range of study participation [and use] thresholds” [1], e.g., N exposed, N consented, N used more than x times, N used more than y weeks, N participants “used” the intervention/comparator at specific pre-defined time points of interest (in absolute and relative numbers per group). Always clearly define “use” of the intervention.

	1	2	3	4	5	
subitem not at all important	○	○	○	○	◉	essential

Clear selection

Does your paper address subitem 16-i? *

Copy and paste relevant sections from the manuscript (include quotes in quotation marks "like this" to indicate direct quotes from your manuscript), or elaborate on this item by providing additional information not in the ms, or briefly explain why the item is not applicable/relevant for your study

“Results from Primary Outcomes are described based on the ITT analysis method (n = 69 participants total; n = 34 in the VRx arm, n = 35 in the Control arm). This more conservative method was chosen to minimize bias and provide a closer representation of the effectiveness of VR-therapy in clinical practice69.”
“The Secondary Outcomes were evaluated only for patients in the VRx arm who received the intervention (n = 29) and are reported per session (n = 47 sessions) unless otherwise indicated.”
“Quality of Life: Responses from 42 participants (VRx arm n = 22, Control arm n = 20) were included in the sentiment analysis of the semi-structured QoL interview after excluding records where insufficient data were collected to calculate a difference score (i.e., cases with missing data due to excluded responses, or where the semi-structured interview was not completed at least once in the Before and After periods).”
Sample size is reported in the title or caption for each table and graph.

16-ii) Primary analysis should be intent-to-treat

Primary analysis should be intent-to-treat, secondary analyses could include comparing only "users", with the appropriate caveats that this is no longer a randomized sample (see 18-i).

	1	2	3	4	5	
subitem not at all important	○	○	○	◉	○	essential

Clear selection

Does your paper address subitem 16-ii?

Copy and paste relevant sections from the manuscript (include quotes in quotation marks "like this" to indicate direct quotes from your manuscript), or elaborate on this item by providing additional information not in the ms, or briefly explain why the item is not applicable/relevant for your study

"Results from Primary Outcomes are described based on the ITT analysis method (n = 69 participants total; n = 34 in the VRx arm, n = 35 in the Control arm). This more conservative method was chosen to minimize bias and provide a closer representation of the effectiveness of VR-therapy in clinical practice69."

17a) For each primary and secondary outcome, results for each group, and the estimated effect size and its precision (such as 95% confidence interval)

Does your paper address CONSORT subitem 17a? *

Copy and paste relevant sections from the manuscript (include quotes in quotation marks "like this" to indicate direct quotes from your manuscript), or elaborate on this item by providing additional information not in the ms, or briefly explain why the item is not applicable/relevant for your study

We chose to report p-values, but not confidence intervals and effect size, due to factors related to our data. The number of events in each outcome were small (see our response to number 10 above), and our results are based on difference scores, making it relatively difficult to interpret the clinical relevance of confidence intervals and effect sizes from our data. Secondary outcomes are provided using descriptive statistics as they were collected for the treatment arm only.

17a-i) Presentation of process outcomes such as metrics of use and intensity of use

In addition to primary/secondary (clinical) outcomes, the presentation of process outcomes such as metrics of use and intensity of use (dose, exposure) and their operational definitions is critical. This does not only refer to metrics of attrition (13-b) (often a binary variable), but also to more continuous exposure metrics such as “average session length”. These must be accompanied by a technical description how a metric like a “session” is defined (e.g., timeout after idle time) [1] (report under item 6a).

	1	2	3	4	5	
subitem not at all important	○	○	○	●	○	essential

Clear selection

Does your paper address subitem 17a-i?

Copy and paste relevant sections from the manuscript (include quotes in quotation marks "like this" to indicate direct quotes from your manuscript), or elaborate on this item by providing additional information not in the ms, or briefly explain why the item is not applicable/relevant for your study

“Participants completed a mean of 1.6 sessions during their stay. Over half of participants (17/29, 58.6%) completed one session, while approximately one fifth of participants (6/29 20.7%) completed two and three (6/29, 20.7%) VR-therapy sessions, respectively. Acceptability of VR: Participants spent an average of 6.8 minutes (SD = 6.6, range = 0-20) viewing VR films during the sessions. Approximately half of participants (15/29, 51.7%) took part in at least one VR session that lasted over 5 minutes, while 11/29 (37.9%) completed only shorter sessions, lasting 1-5 minutes... Sessions that ended early were most often due to the participant’s choice (i.e., patient removed the headset, no distress). Reasons for stopping treatment before the end of each 20-minute session are summarized in Table 4 below.”

17b) For binary outcomes, presentation of both absolute and relative effect sizes is recommended

Does your paper address CONSORT subitem 17b? *

Copy and paste relevant sections from the manuscript (include quotes in quotation marks "like this" to indicate direct quotes from your manuscript), or elaborate on this item by providing additional information not in the ms, or briefly explain why the item is not applicable/relevant for your study

Not applicable/no outcomes were binary.

18) Results of any other analyses performed, including subgroup analyses and adjusted analyses, distinguishing pre-specified from exploratory

Does your paper address CONSORT subitem 18? *

Copy and paste relevant sections from the manuscript (include quotes in quotation marks "like this" to indicate direct quotes from your manuscript), or elaborate on this item by providing additional information not in the ms, or briefly explain why the item is not applicable/relevant for your study

Not applicable/no separate subgroup or adjusted analyses were performed.

18-i) Subgroup analysis of comparing only users

A subgroup analysis of comparing only users is not uncommon in ehealth trials, but if done, it must be stressed that this is a self-selected sample and no longer an unbiased sample from a randomized trial (see 16-iii).

	1	2	3	4	5	
subitem not at all important	○	○	●	○	○	essential

Clear selection

Does your paper address subitem 18-i?

Copy and paste relevant sections from the manuscript (include quotes in quotation marks "like this" to indicate direct quotes from your manuscript), or elaborate on this item by providing additional information not in the ms, or briefly explain why the item is not applicable/relevant for your study

A subgroup analysis comparing only users was not performed for the primary outcomes. However, we report on results of calculations to determine the possibility of a priori bias in the random assignment for participants who received VR therapy vs. those who did not: "To determine the possibility of a priori bias in the random assignment, a Chi-Square Test for Association was run on the pairwise categorical variables (Arm Assignment, Had VR, Age Group, Gender, Dementia Diagnosis, and Cognitive Level). There was no statistically significant association found between any pairs in any distribution method (P>.05).19)"

19) All important harms or unintended effects in each group

(for specific guidance see CONSORT for harms)

Does your paper address CONSORT subitem 19? *

Copy and paste relevant sections from the manuscript (include quotes in quotation marks "like this" to indicate direct quotes from your manuscript), or elaborate on this item by providing additional information not in the ms, or briefly explain why the item is not applicable/relevant for your study

No intervention was provided to the control group. For the treatment group:
"Safety: No serious side effects or adverse events related to having received VR-therapy were observed or reported during the trial. Patients who experienced a serious adverse event during their hospital stay were withdrawn from the study..."

19-i) Include privacy breaches, technical problems

Include privacy breaches, technical problems. This does not only include physical “harm” to participants, but also incidents such as perceived or real privacy breaches [1], technical problems, and other unexpected/unintended incidents. “Unintended effects” also includes unintended positive effects [2].

	1	2	3	4	5	
subitem not at all important	○	○	○	◉	○	essential

Clear selection

Does your paper address subitem 19-i?

Copy and paste relevant sections from the manuscript (include quotes in quotation marks "like this" to indicate direct quotes from your manuscript), or elaborate on this item by providing additional information not in the ms, or briefly explain why the item is not applicable/relevant for your study

“Hardware/Software Technical difficulties: Video on pause, headset and controller out of sync; Number of VRx Sessions (n, %) 2, 4.3%”

19-ii) Include qualitative feedback from participants or observations from staff/researchers

Include qualitative feedback from participants or observations from staff/researchers, if available, on strengths and shortcomings of the application, especially if they point to unintended/unexpected effects or uses. This includes (if available) reasons for why people did or did not use the application as intended by the developers.

	1	2	3	4	5	
subitem not at all important	○	○	○	○	◉	essential

Clear selection

Does your paper address subitem 19-ii?

Copy and paste relevant sections from the manuscript (include quotes in quotation marks "like this" to indicate direct quotes from your manuscript), or elaborate on this item by providing additional information not in the ms, or briefly explain why the item is not applicable/relevant for your study

Qualitative feedback from participants, staff, and family members is described throughout the results section for the secondary outcomes.

DISCUSSION

22) Interpretation consistent with results, balancing benefits and harms, and considering other relevant evidence

NPT: In addition, take into account the choice of the comparator, lack of or partial blinding, and unequal expertise of care providers or centers in each group

22-i) Restate study questions and summarize the answers suggested by the data, starting with primary outcomes and process outcomes (use)

Restate study questions and summarize the answers suggested by the data, starting with primary outcomes and process outcomes (use).

	1	2	3	4	5	
subitem not at all important	○	○	○	◉	○	essential

Clear selection

Does your paper address subitem 22-i? *

Copy and paste relevant sections from the manuscript (include quotes in quotation marks "like this" to indicate direct quotes from your manuscript), or elaborate on this item by providing additional information not in the ms, or briefly explain why the item is not applicable/relevant for your study

Primary Outcomes: “The aims of the current study were to investigate the effects of VR-therapy on BPSD and other related outcomes in PwD admitted to an acute care hospital. Based on encouraging evidence from recent studies58, we hypothesised that by administering VR-therapy with natural virtual environments to people with dementia admitted to an acute care hospital we could help manage BPSD and improve other process measures including falls, QoL and LoS. In line with our predictions, we found that VR-therapy significantly reduced aggressive/violent behavior and loud vocalizations..."
Process Outcomes/Use: “When considering acceptability, safety, and patient experience with VR-therapy, our observational results are overall promising. No serious adverse events occurred that were related to the intervention..."

22-ii) Highlight unanswered new questions, suggest future research

Highlight unanswered new questions, suggest future research.

	1	2	3	4	5	
subitem not at all important	○	○	○	○	◉	essential

Clear selection

Does your paper address subitem 22-ii?

Copy and paste relevant sections from the manuscript (include quotes in quotation marks "like this" to indicate direct quotes from your manuscript), or elaborate on this item by providing additional information not in the ms, or briefly explain why the item is not applicable/relevant for your study

Several are discussed in the “Limitations and Future Directions” section, e.g.:
“This study was subject to several limitations, with potential impact on our ability to answer the research questions, as well as on the ability to generalize the findings. The first, most critical limitation was the early termination of the study due to the COVID-19 pandemic, with a final recruitment of less than half of the target sample size. Follow-up studies with the sufficient sample size required for statistical power will be necessary to determine whether VR-therapy has a significant effect on LoS, falls, and QoL, and BPSD, and to further establish its effectiveness for reducing aggressive behaviors.”

20) Trial limitations, addressing sources of potential bias, imprecision, and, if relevant, multiplicity of analyses

20-i) Typical limitations in ehealth trials

Typical limitations in ehealth trials: Participants in ehealth trials are rarely blinded. Ehealth trials often look at a multiplicity of outcomes, increasing risk for a Type I error. Discuss biases due to non-use of the intervention/usability issues, biases through informed consent procedures, unexpected events.

	1	2	3	4	5	
subitem not at all important	○	○	○	●	○	essential

Clear selection

Does your paper address subitem 20-i? *

Copy and paste relevant sections from the manuscript (include quotes in quotation marks "like this" to indicate direct quotes from your manuscript), or elaborate on this item by providing additional information not in the ms, or briefly explain why the item is not applicable/relevant for your study

“Second, as less than half of the participants completed more than one VR-therapy session, it is difficult to draw conclusions on treatment effectiveness. While this limitation is common among similar studies involving VR-therapy in PwD, it made it more difficult to evaluate the outcomes in general, and it was not possible to measure the impact of treatment dosage or whether a washout period exists after repeated VR exposures..."

21) Generalisability (external validity, applicability) of the trial findings

NPT: External validity of the trial findings according to the intervention, comparators, patients, and care providers or centers involved in the trial

21-i) Generalizability to other populations

Generalizability to other populations: In particular, discuss generalizability to a general Internet population, outside of a RCT setting, and general patient population, including applicability of the study results for other organizations

	1	2	3	4	5	
subitem not at all important	○	○	○	○	◉	essential

Clear selection

Does your paper address subitem 21-i?

Copy and paste relevant sections from the manuscript (include quotes in quotation marks "like this" to indicate direct quotes from your manuscript), or elaborate on this item by providing additional information not in the ms, or briefly explain why the item is not applicable/relevant for your study

Several implications for generalizability of the findings to acute care and other studies of VR involving people living with dementia (applicable in different settings) are described in the "Limitations and Future Directions" section:
"This study was subject to several limitations, with potential impact on our ability to answer the research questions, as well as on the ability to generalize the findings."

21-ii) Discuss if there were elements in the RCT that would be different in a routine application setting

Discuss if there were elements in the RCT that would be different in a routine application setting (e.g., prompts/reminders, more human involvement, training sessions or other co-interventions) and what impact the omission of these elements could have on use, adoption, or outcomes if the intervention is applied outside of a RCT setting.

	1	2	3	4	5	
subitem not at all important	○	○	○	○	◉	essential

Clear selection

Does your paper address subitem 21-ii?

Copy and paste relevant sections from the manuscript (include quotes in quotation marks "like this" to indicate direct quotes from your manuscript), or elaborate on this item by providing additional information not in the ms, or briefly explain why the item is not applicable/relevant for your study

"Nevertheless, a substantial minority of participants in our study (24%) either refused VR or stopped their session after less than one minute. We also observed variability in session length within participants; as one participant described, "Today [is] not a good day. Maybe I'd enjoy tomorrow. Just a bit shaky now..."

OTHER INFORMATION

23) Registration number and name of trial registry

Does your paper address CONSORT subitem 23? *

Copy and paste relevant sections from the manuscript (include quotes in quotation marks "like this" to indicate direct quotes from your manuscript), or elaborate on this item by providing additional information not in the ms, or briefly explain why the item is not applicable/relevant for your study

“ClinicalTrials.gov registration (ID NCT03941119)”

24) Where the full trial protocol can be accessed, if available

Does your paper address CONSORT subitem 24? *

Cite a Multimedia Appendix, other reference, or copy and paste relevant sections from the manuscript (include quotes in quotation marks "like this" to indicate direct quotes from your manuscript), or elaborate on this item by providing additional information not in the ms, or briefly explain why the item is not applicable/relevant for your study

“ClinicalTrials.gov registration (ID NCT03941119)”

25) Sources of funding and other support (such as supply of drugs), role of funders

Does your paper address CONSORT subitem 25? *

Copy and paste relevant sections from the manuscript (include quotes in quotation marks "like this" to indicate direct quotes from your manuscript), or elaborate on this item by providing additional information not in the ms, or briefly explain why the item is not applicable/relevant for your study

“Funding: The study was funded through the Centre for Brain Health and Innovation. The funding bodies had no role in the design of the study, collection, analysis, and interpretation of data, or in writing the manuscript.”

X27) Conflicts of Interest (not a CONSORT item)

X27-i) State the relation of the study team towards the system being evaluated

In addition to the usual declaration of interests (financial or otherwise), also state the relation of the study team towards the system being evaluated, i.e., state if the authors/evaluators are distinct from or identical with the developers/sponsors of the intervention.

	1	2	3	4	5	
subitem not at all important	○	○	○	○	◉	essential

Clear selection

Does your paper address subitem X27-i?

Copy and paste relevant sections from the manuscript (include quotes in quotation marks "like this" to indicate direct quotes from your manuscript), or elaborate on this item by providing additional information not in the ms, or briefly explain why the item is not applicable/relevant for your study

"Conflicts of Interest: None declared."
Funding: The study was funded through the Centre for Brain Health and Innovation. The funding bodies had no role in the design of the study, collection, analysis, and interpretation of data, or in writing the manuscript.
The authors selected the films: "A library of short 360-degree films was assembled based on previous studies and expert consultation..."

About the CONSORT EHEALTH checklist

As a result of using this checklist, did you make changes in your manuscript? *

- ○ yes, major changes
- ◉ yes, minor changes
- ○ no

What were the most important changes you made as a result of using this checklist?

Moved the CONSORT flow diagram down to the results section from the Methods section.

How much time did you spend on going through the checklist INCLUDING making changes in your manuscript *

4 hours

As a result of using this checklist, do you think your manuscript has improved? *

- ( ) yes
- ( ) no
- (•) Other: Minor improvements in clarity.

Would you like to become involved in the CONSORT EHEALTH group?

This would involve for example becoming involved in participating in a workshop and writing an "Explanation and Elaboration" document

- ( ) yes
- (•) no
- ( ) Other:

Clear selection

Any other comments or questions on CONSORT EHEALTH

None.

STOP - Save this form as PDF before you click submit

To generate a record that you filled in this form, we recommend to generate a PDF of this page (on a Mac, simply select "print" and then select "print as PDF") before you submit it.

When you submit your (revised) paper to JMIR, please upload the PDF as supplementary file.

Don't worry if some text in the textboxes is cut off, as we still have the complete information in our database. Thank you!

Final step: Click submit !

Click submit so we have your answers in our database!

Submit

Clear form

Never submit passwords through Google Forms.

This content is neither created nor endorsed by Google. Report Abuse - Terms of Service - Privacy Policy

Google Forms
